# Recent advances in the removal of radioactive iodine by bismuth-based materials

**DOI:** 10.3389/fchem.2023.1122484

**Published:** 2023-01-24

**Authors:** Yuxun Hao, Zhenjiang Tian, Chuanying Liu, Chengliang Xiao

**Affiliations:** ^1^ Institute of Zhejiang University-Quzhou, Quzhou, China; ^2^ College of Chemical and Biological Engineering, Zhejiang University, Hangzhou, China

**Keywords:** spent fuel reprocessing, radioactive iodine, bismuth-based materials, nuclear waste, capture

## Abstract

Nowadays, the demand for nuclear power is continue increasing due to its safety, cleanliness, and high economic benefits. Radioactive iodine from nuclear accidents and nuclear waste treatment processes poses a threat to humans and the environment. Therefore, the capture and storage of radioactive iodine are vital. Bismuth-based (Bi-based) materials have drawn much attention as low-toxicity and economical materials for removing and immobilizing iodine. Recent advances in adsorption and immobilization of vapor iodine by the Bi-based materials are discussed in this review, in addition with the removal of iodine from solution. It points out the neglected areas in this research topic and provides suggestions for further development and application of Bi-based materials in the removal of radioactive iodine.

## 1 Introduction

Nuclear energy is one of the main sources of electrical energy in the world today. It is advantageous in safety, cleanliness, and lower total operating costs than almost all fossil fuels. The main issues that nuclear energy faced are operational safety and the disposal of radioactive waste (Kosaka et al., 2012; Woo, 2013). The released off-gas stream from the reprocessing of spent nuclear fuels and serious nuclear power plant accidents contains a variety of radioactive elements (^99^Tc, ^137^Cs, ^90^Sr, ^129^I, ^131^I, ^3^H^14^C, ^85^Kr, actinides, lanthanides, etc.) (Soelberg et al., 2013; Subrahmanyam et al., 2015; Nandanwar et al., 2016; Riley et al., 2016). Radioactivity is a huge threat to humans and the environment due to its easy diffusion (Ojovan and Lee, 2011; Mowry et al., 2015; Yang et al., 2018; Chabauty et al., 2019). Among these radioactive elements, iodine compounds attract special attention because the iodine is concentrated in the thyroid, and its higher radioactivity can cause damage to the human body in a short time (Grossman et al., 1996; Goldsmith et al., 1999; Grossman et al., 2002; 2003; Lee et al., 2006; Thomas et al., 2009; Sava et al., 2013). The radioactivity is produced by ^129^I, a long-period isotope with a half-life of 1.57 × 10^7^ years (Taylor, 1981; Michel et al., 2005; Zhang et al., 2011; Lin et al., 2019), and ^131^I, a short-lived isotope with a half-life of approximately 8.02 days (Thomas et al., 2009; Lee et al., 2018). In active waste gas, iodine mainly exists in the form of elemental substances, hydroiodic acid, and organic iodine (CH_3_I) (Lin, 1981; Huang et al., 2010; Pillar et al., 2013). The initial form of iodine in spent nuclear fuel is cesium iodide. During the dissolution of spent nuclear fuel, iodine ions will react with free radicals produced by radiation to generate volatile elemental iodine. Part of elemental iodine will also react with organic impurities to generate organic iodine (Wren et al., 1999; Taghipour and Evans, 2000; Moriyama et al., 2010). These volatile iodine species make the separation and safe storage of radioactive iodine a great challenge.

There are two methods to collect gaseous radioisotopes: the wet scrubbing and the solid phase adsorption. The wet scrubbing includes Mercurex, Iodox, electrolytic and caustic scrubbing (Mailen and Horner, 1976; Horner et al., 1977; Riley et al., 2016). In these processes, the radioactive iodine species are chemically reacted with the liquid, whereas a large amount of secondary waste is produced (Riley et al., 2016). Compared with wet scrubbing, solid phase adsorption has attracted more attention because of its low cost, simple operation, and high adsorption efficiency (Munakata et al., 2003). Many kinds of materials have been applied in the adsorption of vapor radioactive iodine, including activated carbon (Deitz, 1987; Chien et al., 2011), graphene-based materials (Scott et al., 2015), zeolites (Chapman et al., 2010; Nenoff et al., 2014; Pham et al., 2016; Abney et al., 2017; Nan et al., 2018), metal-organic frameworks (MOFs) (Sava et al., 2012; Zhang et al., 2019; Miensah et al., 2022), covalent-organic frameworks (COFs) (Wang et al., 2018; Wang and Zhuang, 2019; Yang et al., 2019), layered double hydroxide-based materials (Ma et al., 2014; Lin et al., 2019), and aerogels (Subrahmanyam et al., 2015). In the adsorption of radioactive iodine, loading capacity and adsorption efficiency (separation capacity) are the main criteria for evaluating solid adsorbents, along with the stability under the harsh conditions of radioactive gas. For example, the activated carbon, especially loaded with KI/TEDA, has a strong iodine adsorption capacity due to its high porosity and large specific surface area (Deuber, 1986; Deitz, 1987; Pires et al., 2001; Ampelogova et al., 2002; Gonzalez-Garcia et al., 2013; Herdes et al., 2013; Zhou et al., 2014; Nandanwar et al., 2016). However, the thermal stability of activated carbon is poor, and its adsorption capacity is diminished at the operating temperature of spent fuel processing. Silver-based materials have been widely studied in iodine capture because silver can react with iodine to generate insoluble AgI (Kikuchi et al., 1978; Kindel et al., 1993; Sakurai and Takahashi, 1994; Funabashi et al., 1995; Modolo and Odoj, 1997; Mineo et al., 2002; Mineo et al., 2003; Takeshita and Azegami, 2004; Tanabe et al., 2010; Kulyukhin et al., 2012). Although silver-based materials have excellent performance in capturing ability and stability, the high cost and toxic of silver make it necessary to explore alternatives.

In recent years, Bi-based materials have received attentions from researchers due to their environmental and economic advantages including, 1) the toxicity of bismuth is relatively low, no occupational poisoning caused by bismuth or its compounds has been reported; 2) Bismuth is extremely weak in radioactivity with a half-life of about 1.9 × 10^19^ years; 3) bismuth is much cheaper than silver. In mechanism, bismuth can react with iodine to form BiI_3_. In addition, Bi_2_O_3_ and Bi_2_S_3_ can also react with iodine (Liu et al., 2014; Cordova et al., 2020). In an aqueous solution, the formation of products (Bi_x_O_y_I_z_) depends on the combined effects of the molar ratio of bismuth and iodine, temperature, pH, etc., (Kodama, 1992; Krumhansl and Nenoff, 2011).

Up to now, various types of Bi-based materials have been developed. Tesfay Reda et al. (2021a) divided the Bi-base materials in iodine capture into two categories: the materials applied in the capture of aqueous iodine and in the capture of vapor iodine. The Bi-based materials for capturing aqueous iodine were emphatically summarized. Ranjan et al. (2020) summarized the capture of pollutants in water by Bi-based materials. The study by Moore et al. (2020) mentioned four Bi-based compounds for iodine capture. The Bi-based materials were also summarized in an overview of metal Oxide-based materials (Muhire et al., 2022). Recently, the works about capturing vapor iodine by Bi-based materials is growing rapidly. Still, there is a lack of systematic study of these works. The main purpose of this review is to summarize recent advances in Bi-based materials for capturing radioactive iodine, especially the vapor iodine. Besides, the immobilization of radioactive iodine by Bi-based materials is also discussed. It aims to provide methods and suggestions for developing novel Bi-based iodine adsorption materials.

## 2 Vapor iodine capture

**FIGURE 1:**
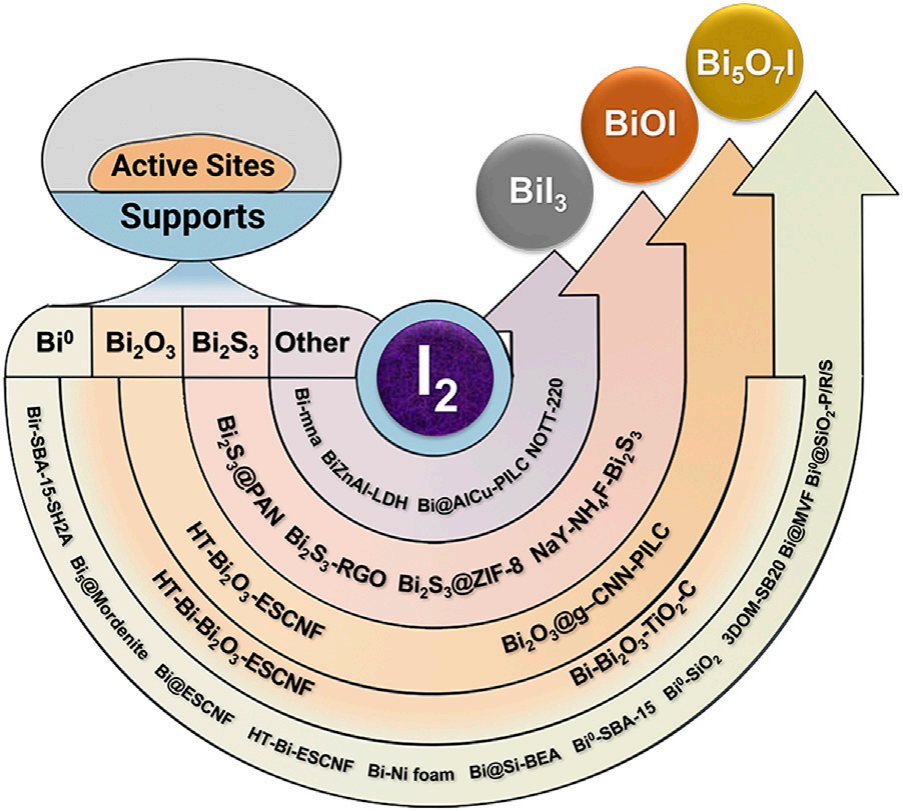
Overview of Bi-based materials for gaseous iodine capture.

Gaseous iodine capture materials are mainly composed of active sites and carriers. The active sites capture iodine by chemical adsorption (like bismuth), and the carriers provide physical adsorption and load the active sites. Various materials have been developed to capture vapor radioactive iodine, and many of them were commercialized. Some of them, such as zeolites, carbon materials, MOFs, and some other materials, were used as carrier for bismuth and its compounds (shown in Figure 1).

### 2.1 Capture mechanism

#### 2.1.1 Carriers

The mechanism of most Bi-based materials capturing iodine composed by the primary chemical adsorption and the secondary physical adsorption. In the solid adsorbent, the main function of carriers is dispersing active sites. Some carriers (molecular sieves, carbon materials, organic polymers, etc.) also exhibit a certain degree of physical adsorption capacity due to their rich microporous structures. However, in the process of loading bismuth by impregnation, the microporous structure restricts bismuth from entering the materials, and the smaller pores are blocked which will decrease the specific surface area and increase the average pore diameter. On the contrary, mesoporous and macroporous structures have almost no physical adsorption capacity. The loading compounds are easier to enter the material, making the specific surface area and pore diameter smaller (Chang et al., 2022a). The observation of Bi^0^ nanoparticles by scanning electron microscopy (SEM) and transmission electron microscopy (TEM) (Tian et al., 2021a; Xian et al., 2022a; Tian et al., 2022) verified the above phenomena.

#### 2.1.2 Active sites

Iodine reacts chemically with bismuth to mainly generate BiI_3,_ although other Bi_x_O_y_I_z_ compounds can be formed (Yang et al., 2015; Yang et al., 2016; Zou et al., 2019; Al-Mamoori et al., 2020). Bi_2_O_3_ and Bi_2_S_3_ also can react with iodine to form stable compounds. These materials’ adsorption mechanisms for iodide can be represented by the following equations (Dinh et al., 2020; Zou et al., 2021; Chen et al., 2022). In addition, bismuth exists in other forms of Bi(Ⅲ) in some materials (Dinh et al., 2020; Tesfay Reda et al., 2021b; Jung et al., 2021; Qin et al., 2022).

$$Bi+1.5I_{2}={BiI}_{3}$$

$${2Bi}_{2}O_{3}+2I_{2}=4BiOI+O_{2}$$

$${Bi}_{2}S_{3}+3I_{2}+3O_{2}={2BiI}_{3}+3{SO}_{2}$$

$${Bi}_{2}S_{3}+3I_{2}={2BiI}_{3}+3S$$

The change of the bismuth state in these materials can be detected by powder X-ray diffraction (PXRD) and X-ray photoelectron spectroscopy (XPS). PXRD pattern can simply indicate the existence of Bi^0^ (2θ = 22.5, 27.2, 38.0, 39.7, 46.1, 46.7, 48.1, 55.8, 55.5, 55.8, 62.7, 64.6, 71.0, and 72.1°; PDF No. 85-1329), Bi_2_O_3_ (2θ = 27.9, 31.8, 32.8, 46.2, 46.9, 54.3, 55.5, 57.8, and 74.5°; PDF No. 78-1793), Bi_2_S_3_ (2θ = 22.4, 24.9, 28.6, 31.8, 32.9, 33.9, 45.5, and 46.4°; PDF No. 17-0320) particles loaded on the material before adsorption and the generation of BiI_3_ (2θ = 12.8, 14.3, 25.7, 27.0, 35.3, 39.2, 41.5, 43.6, 46.1, 50.4, 53.0, 55.6, 58.4, 67.2, 69.5, 71.8, and 72.9°; PDF No. 74-0457) and BiOI (2θ = 29.7, 31.7, 37.0, 39.4, 43.8, 45.4, 51.6, and 55.2°; PDF No. 73-2062) after adsorption. XPS spectra analysis is used to investigate the change in the valence states of bismuth. Due to measurement errors and manual calibration, the peak positions in different reports may be slightly different. The binding energy of Bi^0^ 4f was about 157.2 and 162.6 eV (Xian et al., 2022b). The binding energy of Bi(Ⅲ) 4f is affected by its chemical environment. The duals peaks of Bi@Si-BEA and I-Bi@Si-BEA (Tian et al., 2022) at 159.0, 164.6 eV and 158.9, 164.3 eV were attributed to Bi_2_O_3_ and BiI_3_, while another work deconvolved the characteristic peaks into four peaks (Tesfay Reda et al., 2022). The duals peaks of Bi_2_S_3_@ZIF-8 (Chen et al., 2022) at 158.5 and 163.8 eV were attributed to Bi_2_S_3_. Compared to BiI_3_, the electronegativity of oxygen in BiOI was slightly higher than iodine, resulting in a slight increase in the binding energy of Bi 4f (Tian et al., 2021a). The analysis of I 3d peaks also varied in different works. For example, Qin et al. (2022) assigned the peaks at 618.8, 630.3, 619.6, and 631.1 eV to I^−^, I_3_^−^, I_2_, and I_5_^−^, respectively. The dual peaks in Bi_2_O_3_@g-CNN-PILC were deconvoluted into four peaks at 618.87, 620.57, 630.37, and 631.8 eV which were attributed to I_3_^−^ and I_5_^−^. Tian et al. (2022) assumed that the two peaks at 619.3 eV and 630.8 eV could be attributed to I^−^. The variation in I 3d peaks may be caused by the adsorption form of iodine in different carriers.

In several reports, the distinct lattice stripes could be observed in the high-resolution transmission electron microscopy (HRTEM) images. The lattice spacing of 0.328, 0.320, 0.31, 0.330, 0.30, and 0.256 nm corresponded to the planes of Bi^0^(Xian et al., 2022a; Xian et al., 2022b; Tian et al., 2022), Bi_2_O_3_(Tesfay Reda et al., 2022), Bi_2_S_3_(Jiang et al., 2022), BiI_3_(Xian et al., 2022a; Tian et al., 2022), BiOI (Qin et al., 2022), and I_2_(Xian et al., 2022a), respectively. Besides, Fourier transform infrared spectroscopy (FT-IR), Raman spectroscopy, and nuclear magnetic resonance (NMR) were also applied in the analysis of iodine adsorption by bismuth-based materials.

### 2.2 Bismuth-based Materials

#### 2.2.1 Zeolites or molecular sieves

Zeolites are the microporous aluminosilicate solids, which are the most typical molecular sieves. Their covalent network structures are formed by linking the corner oxygen atoms of AlO_4_ and SiO_4_ tetrahedra. Due to their regular pore structures, acid resistance, high hydrothermal stability, and cation exchange capacities, zeolites are effective materials for adsorbing elemental iodine and its compounds. Several Ag-exchanged zeolites with different structures have been studied for this application, including mordenite (MOR) (Choi et al., 2001; Verger et al., 2001; Choi et al., 2003; Inagaki et al., 2008; Chapman et al., 2010; Zhao et al., 2011; Aspromonte et al., 2013; Soelberg et al., 2013; Nenoff et al., 2014; Bruffey et al., 2016; Chibani et al., 2016; Riley et al., 2016; Zakirova et al., 2017), NaX zeolite (Cheng et al., 2015), NaY zeolite, ZSM-5, ZSM-11 (Chebbi et al., 2017a; Chebbi et al., 2017b), ferrierite and β zeolite.

Compared with silver-exchanged zeolite, bismuth-exchanged zeolite has rarely been applied in the adsorption of vapor iodine. Al-Mamoori et al. (2020) developed bismuth-doped mordenite (Bi_5_@Mordenite). After being exposed to iodine at 200°C for 6 h, its iodine adsorption capacity could reach a maximum of 538 mg I_2_ per gram adsorbent (hereinafter referred to as mg/g), which doubled the iodine adsorption capacity of the raw zeolite (214 mg/g) and Ag@Mordenite (275 mg/g). The SEM and XPS results showed that the surface area and pore volume of zeolite increased due to the dealumination of zeolite in the process of loading bismuth in nitric acid. It is indicated that Bi-exchanged mordenite could be an alternative to Ag-exchanged mordenite for the capture of iodine.

**FIGURE 2:**
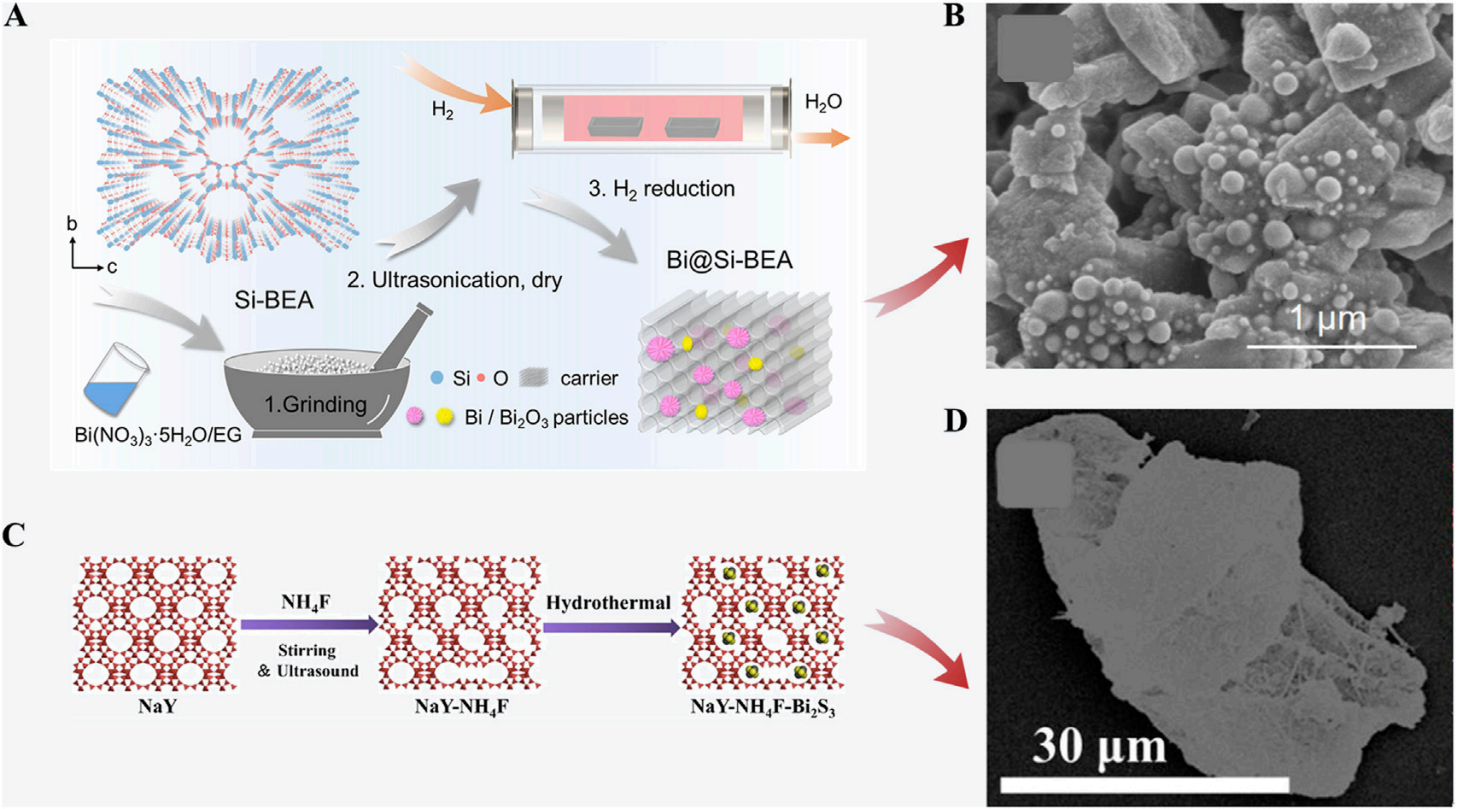
**(A)** Preparation of Bi@Si-BEA; **(B)** SEM image of Bi@Si-BEA (Tian et al., 2022); **(C)** Preparation of NaY-NH_4_F-Bi_2_S_3_; **(D)** Mapping image of NaY-NH_4_F-Bi_2_S_3_(30%) (Jiang et al., 2022).

In industrial processes, the humidity of radioactive iodine off-gas is relatively high which could diminish the iodine capture ability of most zeolites. Tian et al. (2022) introduced the all-silica beta zeolite to improve the hydrophobic properties. A bismuth-modified all-silica beta zeolite (Bi@Si-BEA) (Figures 2A, B) was synthesized through a wetness impregnation process followed by ultrasonication and H_2_ reduction. The total iodine adsorption capacity can reach 650 mg/g at approximately 120°C. The synthesis process was scaled up for possible industrial applications. However, the mechanisms of effects from water vapor or other substances in off-gas require more investigation. Hydrophobic modification of the zeolite is another way to reduce the negative effect of water vapor. Recently, Jiang et al. (2022) developed a hydrophobic zeolite (NaY-NH_4_F-Bi_2_S_3_) containing Bi_2_S_3_ by NH_4_F solution etching and a hydrothermal method (Figures 2C, D). The maximum adsorption capacity of NaY-NH_4_F-Bi_2_S_3_ (30 wt%) at equilibrium is 491 mg/g. The adsorption capacity and equilibration time of the zeolite (50 wt%) both decreased after granulation.

Bismuth-modified SBA-15 is one of the relatively well-studied molecular sieves. Yang et al. (2015) first applied bismuth-doped mesoporous silica (SBA-15) in the field of iodine adsorption and long-term storage. Bismuth was incorporated into the SBA-15 material by previously modifying the silica surface with thiol groups and subsequent thermal treatment that led to the formation of a Bi_2_S_3_ phase. The as-synthesized sorbents could effectively capture iodine gas with the maximal loading capacity of 540 mg/g, which was attributed to the strong reaction tendency of bismuth sulfide with iodine gas, as well as elevated specific surface area and porosity of SBA-15. Furthermore, a chemically durable iodine-loaded material was made with a facile post-sorption process, during which the iodine-incorporated phase was effectively changed from BiI_3_ to chemically durable Bi_5_O_7_I.

Furthermore, the bismuth-embedded SBA-15 is examined as a vapor iodine filtration material at a higher temperature up to 250°C (Kang et al., 2020). These Bi-based SBA-15 materials with high iodine capacities are a promising alternative for radioiodine storage, principally thanks to their high thermal stability. The authors also tried to reduce costs and scale up synthesis by optimizing the element ratio of raw materials and reaction conditions. However, the high price and the cumbersome preparation steps have limited this effort and the material’s further use.

**FIGURE 3:**
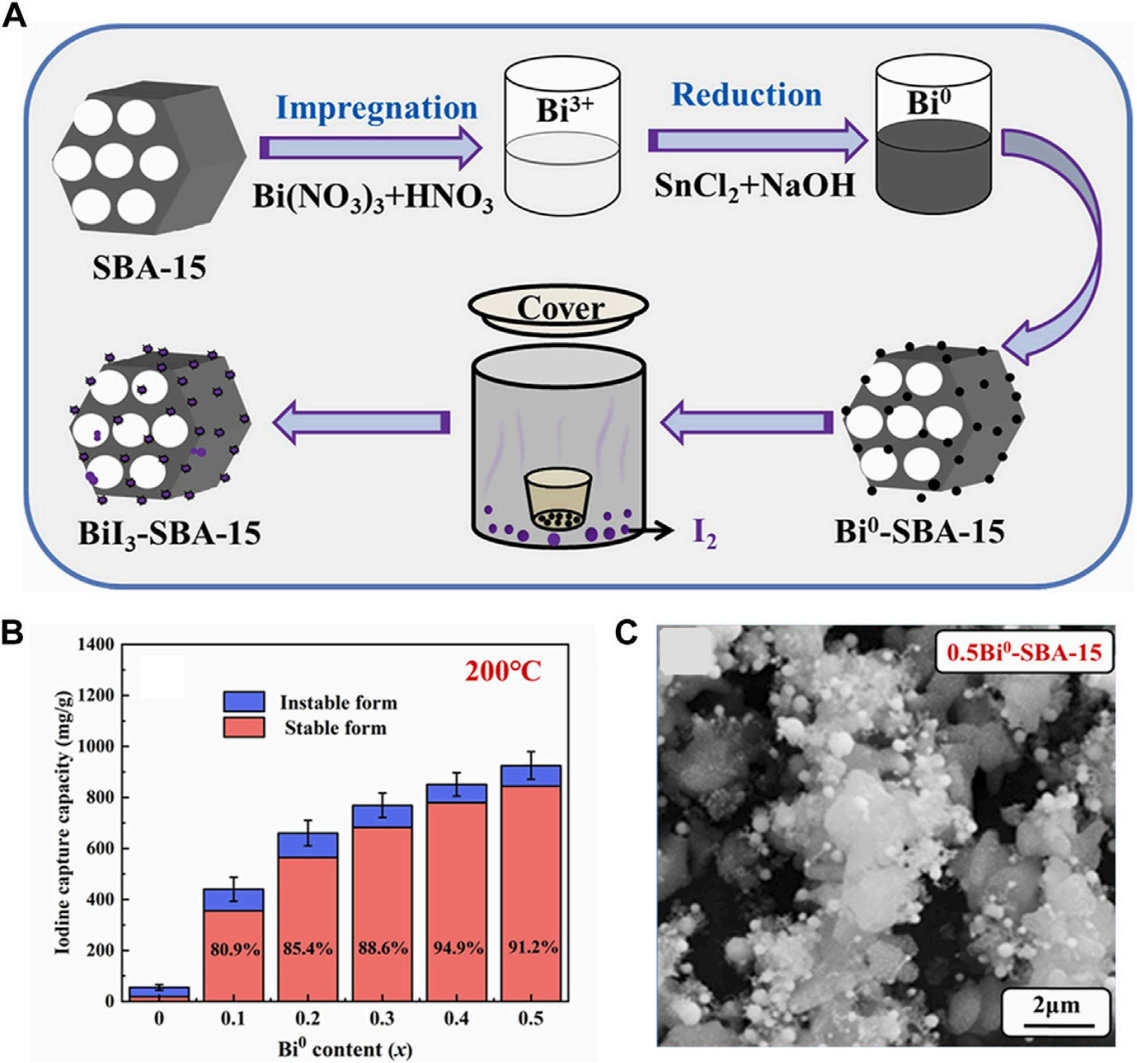
**(A)** Preparation and Iodine Adsorption of Bi^0^-SBA-15; **(B)** Capture Capacity of Iodine at 200°C; **(C)** SEM image of 0.5Bi^0^-SBA-15 (Xian et al., 2022a).

Recently, Xian et al. (2022a) improved the impregnation reduction method to facilely fabricate Bi^0^-SBA-15, with Bi (NO_3_)_3_∙5H_2_O as bismuth source and SnCl_2_∙2H_2_O as reductant (Figure 3). The reaction is represented by the following equation. The bismuth was loaded on the surface of SBA-15 in the form of flocculent and spherical nanoparticles, which provides abundant active sites. The capture capacity was up to 925 mg/g at 200°C within 60 min (Figure 3B). They also scaled up the synthesis with the same route to get a Bi^0^-SiO_2_ which had a capture capacity of 1019 mg/g at 200°C (Xian et al., 2022b). Compared with SBA-15, commercial SiO_2_ is affordable, easy to be scaled up, and time-saving but has a smaller specific surface area. The adsorption capacity after granulation needs to be further investigated.

$$3Sn{Cl}_{2}+2Bi{\left({NO}_{3}\right)}_{3}+18NaOH=2{Bi}^{0}\left(s\right)+6NaCl+6NaNO_{3}+3{Na}_{2}SnO_{3}+9H_{2}O(l)$$

Ding et al. (2023) prepared Bi^0^ modified SiO_2_ with different morphologies (Bi^0^@SiO_2_-P/R/S) by a similar method with the different proportion of raw materials. The maximum capture capacity was 960 mg/g for Bi^0^@SiO_2_-P, and the minimum was 770 mg/g for Bi^0^@SiO_2_-S, as the temperature increased from 75°C to 200°C where the morphologies had changed. They found that more ordered, straight-connected, and open pores are conducive to physical adsorption, while disordered structures are more thermally stable.

Chang et al. (2022a), Chang et al. (2022b) also prepared three-dimensional ordered macropores silica (3DOM-SiO_2_) aerogels with tetraethyl orthosilicate (TEOS), the main raw materials of SBA-15, by the solvent template method, and doped Bi in the materials on this basis. The macropores (about 125 nm) in 3DOM effectively reduce gas diffusion resistance, and the micropores still work as the adsorption pores. The iodine capture capacity was about 696 mg/g at 200°C. The effects of calcination rate and calcination temperature on the structure of materials were also investigated. The lower calcination rate is conducive to the formation of micropores. The higher calcination temperature is not only conducive to the removal of water but also leads to the reduction of micropores due to the crystallization of silica.

#### 2.2.2 Carbon materials

In 2020, Chee et al. (2020) developed a bismuth-decorated electrospinning carbon nanofiber to capture radioactive iodine. At 200°C, the capture capacity of Bi@ESCNF for iodine gas could reach 559 mg/g, thanks to the chemical reaction between Bi and iodine to generate stable BiI_3_. Bi@ESCNF is a macroscopic membrane morphology more suitable for nuclear industry adsorption than another powdered adsorbent. By adding a hydrothermal step based on electrospinning, pre-oxidation, and carbonization process, Tian et al. (2021a) developed three new electrospinning carbon nanofibers (HT-Bi_2_O_3_-ESCNF, HT-Bi-Bi_2_O_3_-ESCNF, and HT-Bi-ESCNF). Under 200°C, they reached adsorption capacities of 364 mg/g, 461 mg/g, and 732 mg/g, respectively. SEM and TEM results showed that most Bi and Bi_2_O_3_ nanoparticles were uniformly and densely anchored on the surface of carbon fibers compared with Bi@ESCNF. Combining PXRD, SEM, TEM, BET, XPS, Raman spectra, and TGA characterizations indicated a small number of polyiodide anions formed by charge transfer between carbon fibers. Iodine molecules were firmly trapped on the carbon fibers, except for the BiI_3_ and BiOI which were generated by the chemical reaction between Bi/Bi_2_O_3_ and I_2_.

**FIGURE 4:**
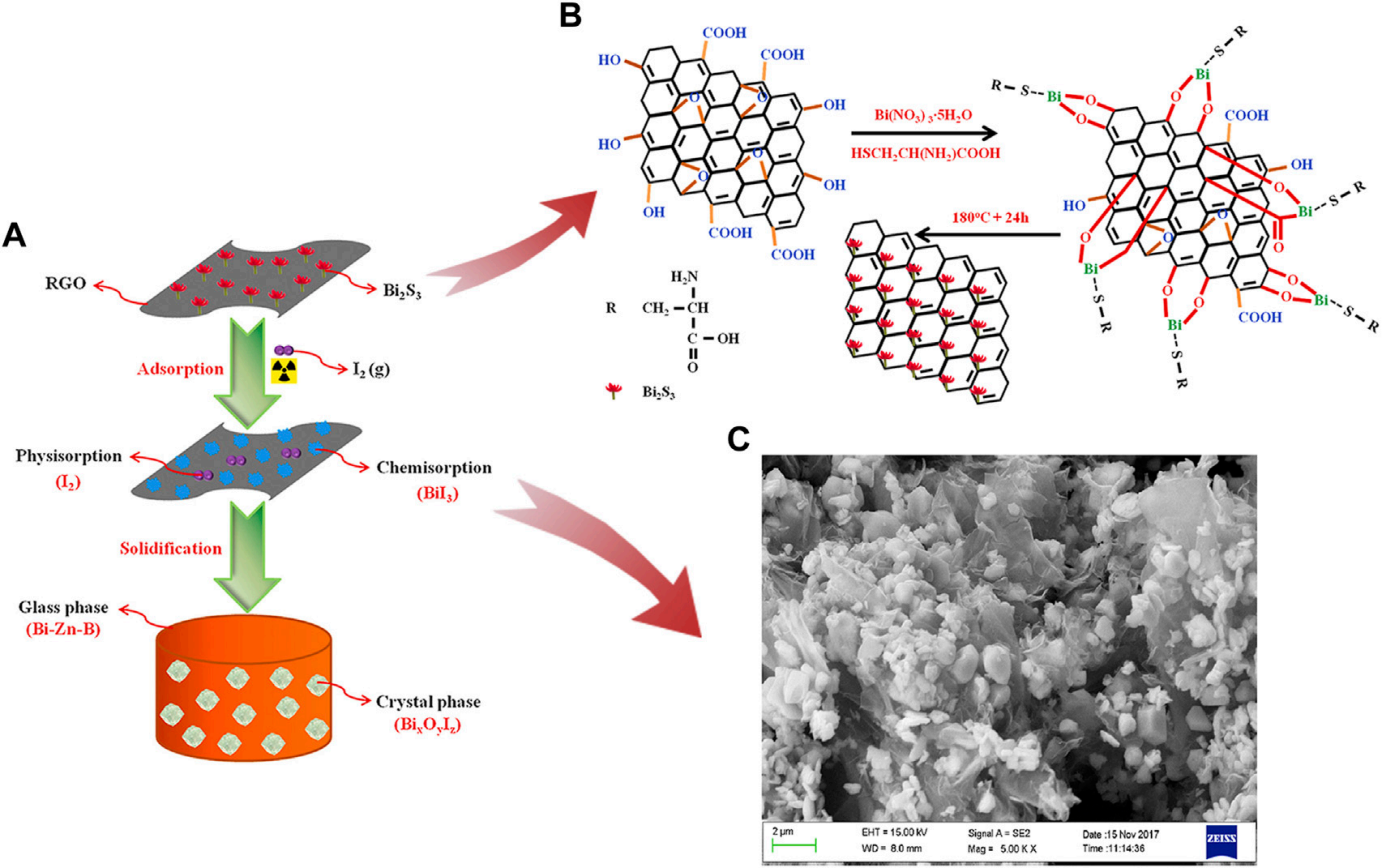
**(A)** and **(B)** Preparation, Iodine Adsorption and Immobilization of Bi_2_S_3_-RGO; **(C)** FESEM image Bi_2_S_3_-RGO-I (Zou et al., 2021).

**FIGURE 5:**
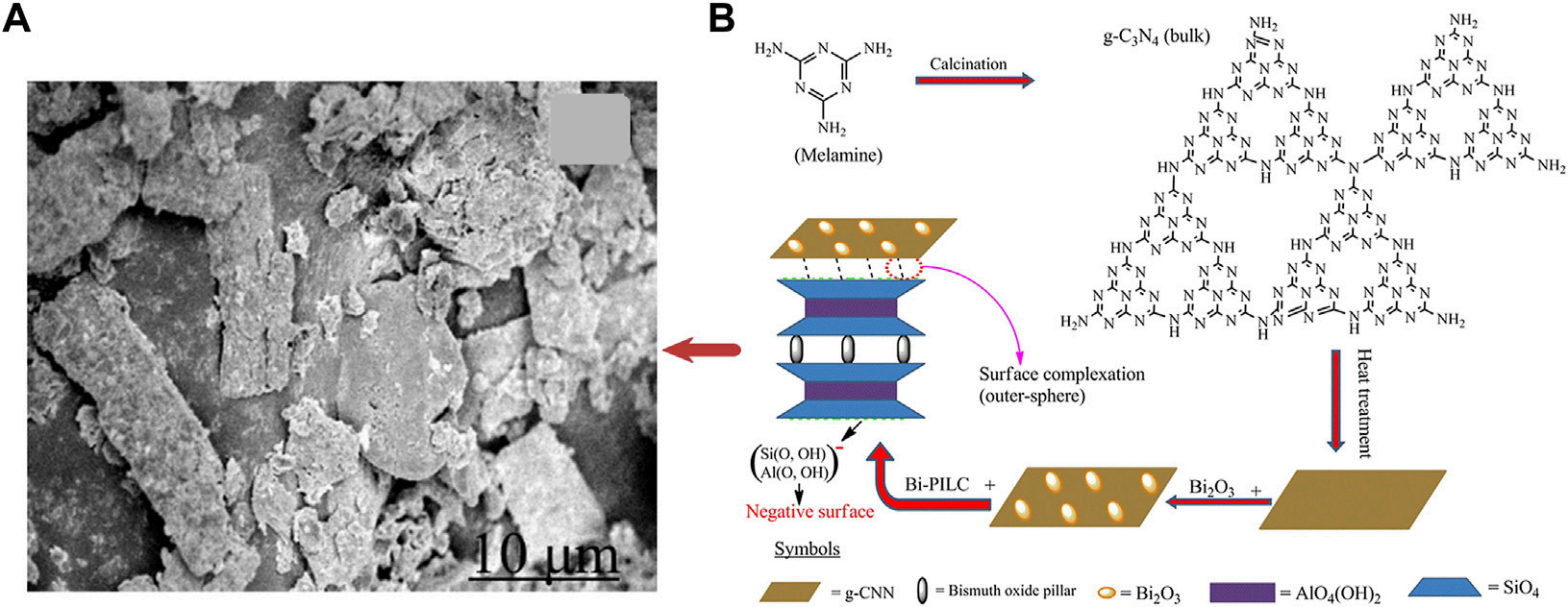
**(A)** SEM image Bi_2_O_3_@g-CNN-PILC; **(B)** Preparation of Bi_2_O_3_@g-CNN-PILC (Tesfay Reda et al., 2022).

In another research, Zou et al. (2021) reported a composite with high iodine capture capacity. The Bi_2_S_3_ reduced graphene oxide (RGO) was produced *via* a solvothermal method which was shown in Figure 4. The adsorption capacities of iodine could reach 1042.8 mg/g at 200 °C for 2 h under a static air atmosphere. Graphitic carbon nitride (g-C_3_N_4_) or graphitic carbon nitride nanosheets (g-CNN) can be easily obtained by modifying melamine, which is eco-friendly and cheap. Tesfay Reda et al. (2022) synthesized Bi_2_O_3_@g–CNN and added pillared interlayered clays (PILC) to increase stability, as shown in Figure 5. The product, Bi_2_O_3_@g–CNN-PILC, had an iodine capture capacity of 830 ± 44 mg/g at 100°C within 8 h. Liu et al. (2022) prepared a bismuth-based porous carbon material (Bi@MVF) by directly carbonizing ZIF-8. Bismuth particles are uniformly embedded and allotted on the porous carbon network, and the capture capacity was up to 1560 mg/g at 120°C for 4 h.

#### 2.2.3 MOFs

In the past 20 years, various studies have been carried out on metal-organic frameworks (MOFs) owing to their extremely high specific surface areas (up to 10^4^ m^2^ g^−1^), high compositional flexibility, and selective gas absorption (Pei et al., 2014). The most commonly studied MOFs for the capture of iodine were the zeolitic imidazolate framework Zn(2-methylimidazolate)_2_ (ZIF-8) and Cu_3_(benzene-1,3,5-tricarboxylate)_2_(H_2_O)_3_ (Cu-BTC) (Sava et al., 2011; Bennett et al., 2013; Hughes et al., 2013; Yuan et al., 2016; Sava Gallis et al., 2017; Chebbi et al., 2018).

**FIGURE 6:**
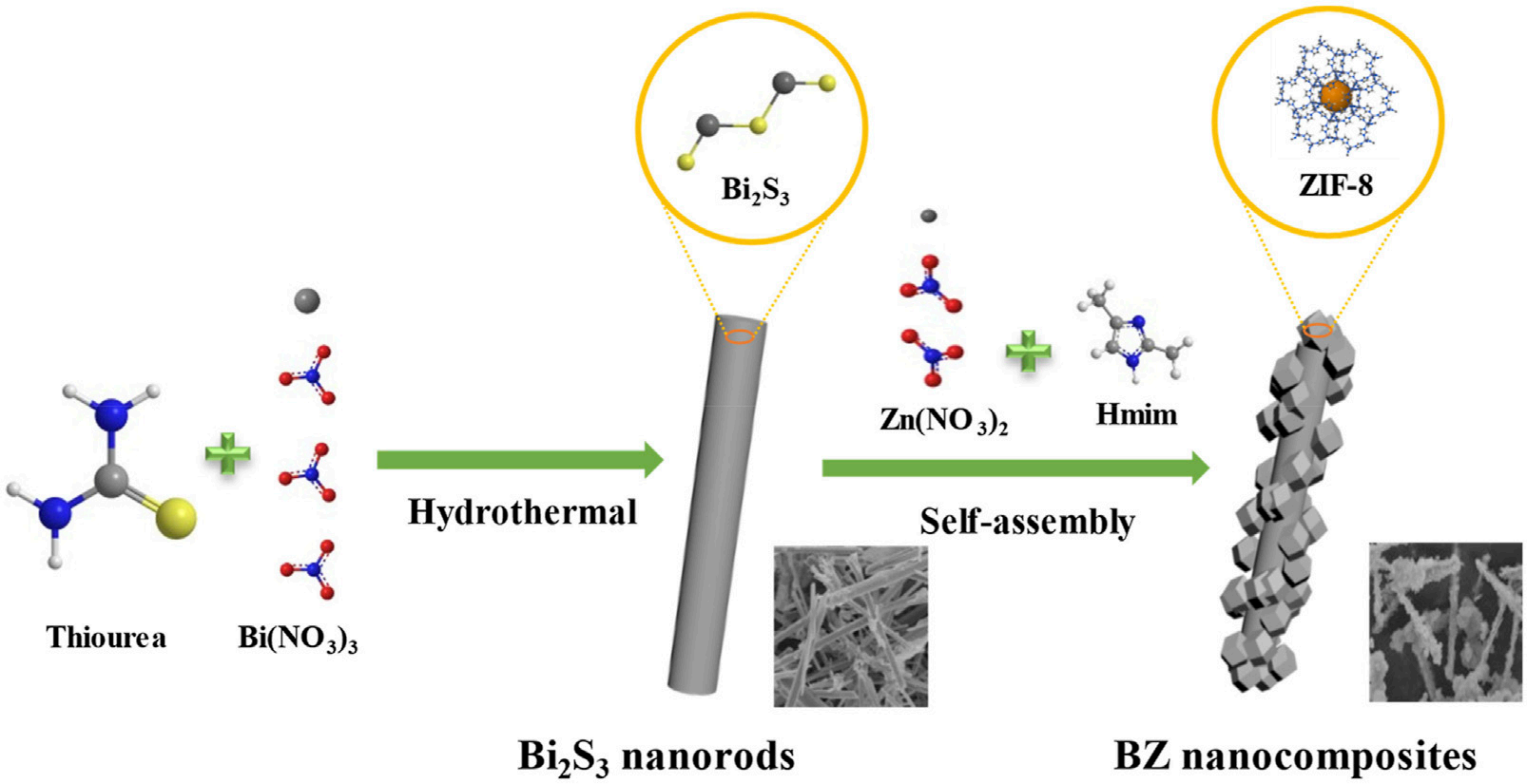
Fabrication of BZ core-shell nanocomposites (Chen et al., 2022).

Chen et al. (2022) modified Ding’s preparation process (Ding et al., 2018) and synthesized Bi_2_S_3_@ZIF-8 (BZ), as shown in Figure 6. The ZIF-8 nanocrystals grow uniformly on the surface of Bi_2_S_3_ nanorods, which inhibits the aggregation of Bi_2_S_3_ nanorods during adsorption. The maximum iodine adsorption capacity of BZ-4 reached 2637.0 mg/g. However, the adsorption equilibrium time was much longer than that of zeolite materials (6 h), and the temperature was only 76.85°C, which was lower than the temperature in the industrial process (about 140°C).

Besides, some MOFs with bismuth ions directly act as connectors in the framework have been reported. They are mostly used for photocatalysis and gas adsorption. Jung et al. (2021) found that 2-mercapto-nicotinic acid (2-mna) is the best candidate for iodine capture out of several composite MOFs. Bi-mna provided strong chemisorption between bismuth and iodine, resulting in high thermal stability. Though the Bi-mna exhibited a lower adsorption capacity than ZIF-8 due to lower MOF density, it was sufficiently large (700 mg/g) for practical applications. Qin et al. (2022) investigated NOTT-220, a kind of MOF material consisting of biphenyl-3,3′,5,5′-tetracarboxylic acid and Bi^3+^ ions, as an iodine gas adsorbent. The adsorption capacities of iodine increased rapidly in 7 h and finally reached the maximum value of 955 mg/g at 75°C in 27 h.

#### 2.2.4 Other materials

Yu Q et al. (2020) synthesized millimeter-sized spherical bismuth sulfide@polyacrylonitrile (Bi_2_S_3_@PAN) hybrid beads. Under 75°C, the iodine capture capacity of Bi_2_S_3_@PAN hybrid beads could reach 986 mg/g. Compared with most powder materials, the beads exhibit not only high iodine adsorption properties but also easy storage and manipulation.

**FIGURE 7:**
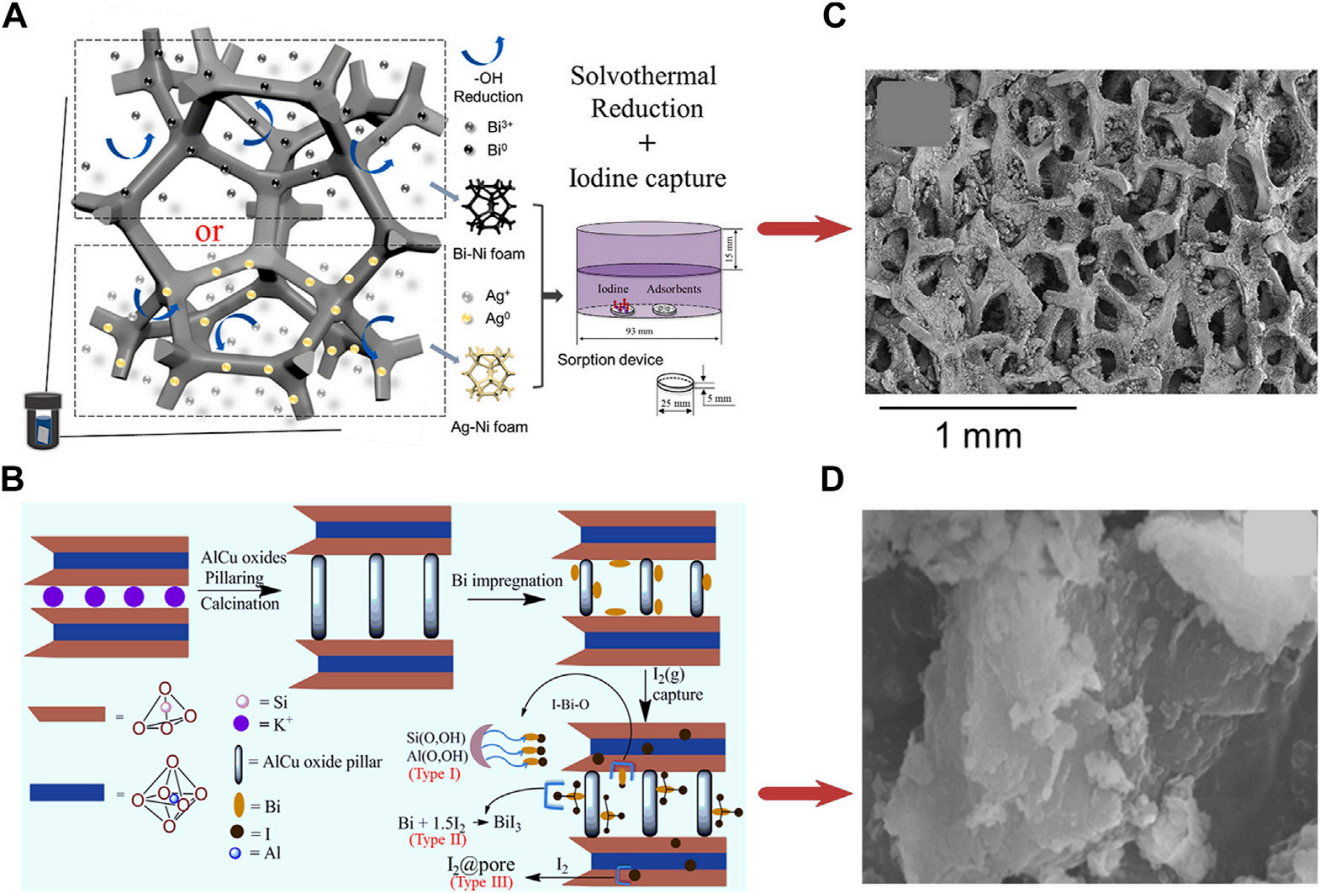
**(A)** Preparation and Iodine Adsorption of Bi-Ni and Ag-Ni foams; **(B)** SEM image of I-Bi-Ni foam (Tian et al., 2021b); **(C)** Preparation and Iodine Adsorption of Bi@AlCu-PILC; **(D)** SEM image of Bi@AlCu-PILC-I (Tesfay Reda et al., 2021b).

Another report conducted by Tian et al. (2021b) developed bismuth and silver functionalized Ni foam composites (Bi-Ni foam and Ag-Ni foam). As illustrated in Figure 7A, Bi-Ni foam shows a higher iodine capture capacity (658 mg/g) but slower adsorption kinetics than Ag-Ni foam (456 mg/g). Due to the more active sites (Bi^0^ or Ag^0^ particles) and the external structure of the Ni foam skeleton, the physically adsorbed iodine is much less than the chemically adsorbed iodine (96% iodine captured in the term of stable form in the I-Bi-Ni foam). Besides, thanks to the nickel skeleton, the material has the advantages of convenient shaping and manipulation.

Zou et al. (2019) developed Bi-Bi_2_O_3_-TiO_2_-C, which was prepared by a sol-gel method for capturing vapor iodine. Bi-Bi_2_O_3_-TiO_2_-C exhibited an iodine adsorption capacity of up to 504 mg/g, which was almost two-fold higher than that of AgX. The authors proposed that majority of iodine was chemically captured in the form of BiI_3_ while a small amount was also physically captured as I_2_. In addition, TiO_2_ contributes to an important portion of the captured iodine.

A bismuth-modified zinc aluminum layered double hydroxide (BiZnAl-LDH) was synthesized *via* co-precipitation by Dinh et al. (2020). The adsorption capacity for iodine reached 433 mg/g. The TGA curves showed that the removal of dehydroxylated hydrotalcite caused another mass loss between the removal of the physically and chemically adsorbed iodine, which is different from other materials.

By impregnating AlCu-oxides pillared Montmorillonite (MMN) with bismuth, Tesfay Reda et al. (2021b) produced Bi-based mesoporous material (Bi@AlCu-PILC), as shown in Figure 7B. AlCu-oxides formed pillars in the gallery of montmorillonite by ion exchange. After calcination, the bismuth was loaded onto these pillars. This single-phase material had high capture capacity (485 ± 54 mg/g under 75°C) and thermal stability.

Several authors have studied chalcogen aerogel containing bismuth as potential adsorbents for iodine, technetium, and uranium trapping. Riley et al. (2013) developed structured aerogels, Co_0.7_Bi_0.3_MoS_4_ (CoBiMoS), which could remove more than 99.0% vapor iodine over the test duration. The adsorption mechanism has also been reported. Chalcogens enclosed in chalcogels are classified as weak Lewis bases according to the hard and soft acids and bases (HSAB) concept (Riley et al., 2011). Consequently, the chalcogens have a high affinity for iodine (I_2_), which is known as a weak Lewis acid. The removal efficiency was governed by the affinity of I_2_(g) and S other than the surface area of chalcogels. This aerogel has the potential for radionuclides removal from solution and gas. However, sulfur compounds could be formed considering the sulfur is part of the material, which will produce secondary pollution. The thermal stability of stored iodine over 150°C has not been examined in this study. However, chalcogels with other adsorbents (Zn_2_Sn_2_S_6_ and Sb_4_Sn_3_S_12_) (Subrahmanyam et al., 2015) have a quite low thermal stability of iodine capture (or the formed iodides). TGA results showed that beyond that temperature, iodine was released progressively.

### 2.3 Summary

The capture capacity of the materials mentioned above is summarized in Table 1. The performance of most materials are better than that of bare adsorbents or Ag-modified adsorbents, which is partly due to the reaction mechanism between bismuth and iodine. However, due to the different thermal stability of raw materials and other reasons, the optimal capture temperature varied widely. At the same time, the volume of the containers used in the static adsorption experiments and the mass of solid iodine added were also different. There are still few studies in this topic, making it difficult to compare the actual properties of different materials objectively. The density of different materials is also dissimilar, resulting in large differences in volume which is important in practical adsorption devices.

**TABLE 1:** Bi-based materials for vapor iodine capture.

Material	Forms of bismuth	Load content (wt%)	Adsorption temperature (°C)	Capture capacity (mg/g)	Approximate equilibrium time (h)	Modification method	Ref.
Bi_5_@Mordenite	Bi_2_O_3_	5	200	538	6	Impregnation, Calcination	Al-Mamoori et al. (2020)
Bi@Si-BEA	Bi^0^	25	160	650	2	Impregnation, H_2_ Reduction	Tian et al. (2022)
NaY-NH_4_F-Bi_2_S_3_	Bi_2_S_3_	30	75	491	6	Etching, Hydrothermal	Jiang et al. (2022)
Bir-SBA-15-SH2A	Bi_2_S_3_	/	200	540	/	Hydrothermal, Impregnation, H_2_ Reduction	Yang et al. (2015)
Bi-SBA-15	Bi_2_S_3_	/	150	−750	/		Kang et al. (2020)
Bi^0^-SBA-15	Bi^0^	25	200	925	1	Impregnation, H_2_ Reduction	Xian et al. (2022a)
			150	1027			
Bi^0^-SiO_2_	Bi^0^	/	200	1019	1	Impregnation, H_2_ Reduction	Xian et al. (2022b)
Bi@ESCNF	Bi^0^	/	200	559	1.5	Electrospinning, Pre-oxidation, Carbonization	Chee et al. (2020)
HT-Bi-ESCNF	Bi^0^	/	200	732	1.5	Electrospinning, Pre-oxidation, Carbonization, Hydrothermal	Tian et al. (2021a)
HT-Bi-Bi_2_O_3_-ESCNF	Bi^0^ Bi_2_O_3_			461	0.75		
HT-Bi_2_O_3_-ESCNF	Bi_2_O_3_			364	0.5		
Bi_2_S_3_-RGO	Bi_2_S_3_	/	200	1042.8	/	Solvothermal	Zou et al. (2021)
Bi_2_O_3_@g–CNN-PILC	Bi_2_O_3_	15	100	830	8	Impregnation, Solvothermal	Tesfay Reda et al. (2022)
Bi-mna	Bi^3+^	/	77–227	−700	/	Solvothermal	Jung et al. (2021)
Bi_2_S_3_@ZIF-8 (BZ)	Bi_2_S_3_	5	77	2637	6	Solvothermal, Mix	Chen et al. (2022)
BiZnAl-LDH	Bi^3+^	/	75	433	/	Mix, Crystallization	Dinh et al. (2020)
Bi_2_S_3_@PAN	Bi_2_S_3_	70	75	986	/	Hydrothermal, Polyacrylonitrile Hybrid	Yu Q et al. (2020)
Bi@AlCu-PILC	Bi^3+^	20	75	485	72	Impregnation	Tesfay Reda et al. (2021b)
Bi-Bi_2_O_3_-TiO_2_-C	Bi^0^ Bi_2_O_3_	/	200	504	<2	Sol-gel, Calcination	Zou et al. (2019)
Bi-Ni foam	Bi^0^	/	200	618	2.5	Solvothermal	Tian et al. (2021b)
3DOM-SB20	Bi^0^	20	200	696	/	Sol–gel, Calcination	Chang et al. (2022a)
NOTT-220	Bi^3+^	/	75	955	27	Heating	Qin et al. (2022)
Bi@MVF	Bi^0^	20	120	1560	6	Carbonization, Impregnation, H_2_ Reduction	Liu et al. (2022)
Bi^0^@SiO_2_-P	Bi^0^	/	960	75	2	Templet Synthesis, Impregnation, H_2_ Reduction	Ding et al. (2023)
			890	130			
			830	200			

In practical industrial processes, the inevitably generated CH_3_I must be removed because of its high toxicity and radioactivity. Compared to silver-based materials, the ability of Bi-based materials to adsorb CH_3_I needs to be further investigated. Besides, many materials’ considerable capture capacities are owing to the large number of active sites on the materials’ surface. It is necessary to study how to test the performance in dynamic adsorption experiments while keeping the performance after granulation.

## 3 Materials stability and immobilization

### 3.1 Materials stability after adsorption

The stability of the materials after adsorption is mainly divided into chemical durability and thermal stability.

Physically adsorbed iodine will desorb over time, especially if the iodine-containing waste is exposed to hot air or solution. A higher ratio of physical adsorption capacity corresponds to the poor chemical durability of waste. Al-Mamoori et al. (2020) evaluated the chemical durability of Bi_5_@Mordenite after adsorption. 30 mg of Bi_5_@Mordenite-I was inserted in 3 ml of deionized water, and 36% of the iodine was released from Bi_5_@Mordenite-I after 24 h of contact. The results demonstrated that Bi_5_@Mordenite is unsuitable for storing radioactive iodine because of its low stability.

In the TGA of Bi-based materials and immobilization tests, BiOI and Bi_7_O_5_I showed better thermal stability. In most experiments, the BiI_3_ decomposed in the range of 200°C–500°C with the reducing of iodine to bismuth ratio, which led to the escape of iodine. The mechanisms can be demonstrated by the following equations (Yang et al., 2016; Zou et al., 2019; Zou et al., 2021). To safely immobilize iodine, additional bismuth must be mixed with the waste of adsorption materials.

$$2{BiI}_{3}+O_{2}=2BiOI+2I_{2}$$

$$\frac{1}{3}BiI_{3}+\frac{7}{3}{Bi}_{2}O_{3}={Bi}_{5}O_{7}I$$

$$BiI_{3}+0.5O_{2}=BiOI+I_{2}$$

$$5BiOI+O_{2}={Bi}_{5}O_{7}I+2I_{2}$$

$$\frac{5}{2}{Bi}_{2}O_{3}+\frac{1}{2}I_{2}={Bi}_{5}O_{7}I+\frac{1}{4}O_{2}$$

The thermal stability of the waste includes that of bismuth-iodine compounds (Bi_x_O_y_I) and supports. Comparing TGA and adsorption results of different materials, under approximately 200°C, BiI_3_ and BiOI are not easily oxidized. The slight weight loss is attributed to the loss of free water and physically adsorbed iodine. Such as Bi_2_S_3_-RGO-I, only 1.5% of weight was lost (the physically adsorbed water and iodine) till 200°C, and the left 83.5% weight was lost from 200°C to 450°C (the chemically adsorbed iodine). The TG-DSC results of Bi-Bi_2_O_3_-TiO_2_-C showed a vast weight loss of 47.97% when heated from 200°C to 300°C, which suggested that the stability of the material was relatively low. The TGA curves showed that after iodine capture, Bi_2_O_3_@g–CNN-PILC (Tesfay Reda et al., 2022) only lost 18.7% of the total weight when heated up to 800°C, which exhibited excellent thermal stability.

### 3.2 Immobilization

For the above reasons, additional bismuth materials are necessary to prevent iodine overflow during immobilization. A study led by Yang et al. (2016) aimed to stabilize iodine confined in Bi-embedded SBA-15. The iodine-containing waste was mixed with low-temperature sintering glasses and Bi_2_O_3_, which were used as binders and stabilizing additives, respectively. A quite satisfactory leaching rate of iodine was measured by a PCT test ranging from 10^−3^ to 10^−2^ g/m^2^ day, which suggested that the glass composite waste form of bismuth embedded SBA-15 could be a potential candidate material for the stable storage of ^129^I. However, the leaching experiment was not performed over an extended duration, and further investigation of stability over a longer period is necessary for better insight. Bi@AlCu-PILC (Tesfay Reda et al., 2021b) was transformed into a more stable form (Bi_5_O_7_I) by post-sorption treatment with Bi_2_O_3_ at 500°C, which had the potential to serve as a long-term disposable iodine waste form.

Some other studies have explored the use of bismuth-containing glass powder for solidification and sintering after adsorption. In these studies, bismuth was added in the immobilization and sintering process to prevent iodine’s escape, while majority of the adsorbents were silver-based materials. Chabauty et al. (2019) synthesized a materials of bismuth-silver phosphate glasses (AgPO_3_-Bi), in which 12 wt% of iodine was chemically immobilized. Combining ^31^P MAS NMR, Raman spectroscopy, and X-ray absorption spectroscopy, it was found that the decomposition of Bi_2_O_3_ resulted in the formation of new bonds in the glass mixture with additional bismuth. Garino et al. (2011) mixed low-temperature sintering bismuth-silicon-zinc-oxide glass powder with AgI or AgI-Mordenite to create the artificial iodine waste form. According to the TGA results, the AgI-Bi-Si-Zn-oxide glass showed no weight loss when heated to 550°C at 10°C/min, while the mass loss of AgI-Mordenite was 18.2% when heated at 20°C/min to 600°C. In studies by Wei et al. (2020), He et al. (2022) and other researchers (Liu et al., 2021; Wei et al., 2021; Wu et al., 2021; Yan et al., 2021; Wu et al., 2022), B_2_O_3_-Bi_2_O_3_-ZnO composites were used for immobilization of iodine waste in the form of AgI with low sintering temperature. Yang et al. (2021); Yang et al. (2022) prepared a Bi-based silica ceramic composite (Si-Cs_3_Bi_2_I_9_ perovskite) for iodine immobilization. Compared to other materials, this material had a high bismuth to iodine ratio but low stability. There was a significant loss of iodine and bismuth in the leaching experiment at room temperature or 90°C after 14 days.

## 4 Aqueous iodine capture

### 4.1 Capture mechanism and solubility

The adsorption mechanisms for aqueous iodide and iodate can be represented by the following equations (Liu et al., 2014; Xiong et al., 2017). According to thermodynamics, bismuth materials’ capture capacity is less affected by the other coexisting anions due to the existence of Bi-O-I and Bi-O-(IO_3_) bonding.

$${Bi}_{2}O_{3}+H_{2}O+2I^{-}=2BiOI+2{OH}^{-}$$

$$2{Bi}_{2}O_{3}+2I^{-}+H_{2}O={Bi}_{4}O_{5}I_{2}+2{OH}^{-}$$

$${Bi}_{6}O_{7}+6{IO}_{3}^{-}+3H_{2}O+O_{2}\to 6BiO\left({IO}_{3}\right)+6{OH}^{-}$$

Different from the capture of vapor iodine, the formation of Bi_x_O_y_I_z_ depends on the bismuth to iodine ratio in the solution. By leaching experiments, Krumhansl and Nenoff (2011) showed that the stability of Bi_5_O_7_I was better than BiOI as the bismuth to iodine ratio increased. Similar to the immobilization results, Bi_5_O_7_I is more stable than other Bi_x_O_y_I_z_ compounds at a higher temperature (Kodama, 1992). However, at a lower temperature, some other stable iodine waste form of BiOI (Krumhansl and Nenoff, 2011; Ng and Fan, 2016; Han et al., 2019; Wang et al., 2020; Yang et al., 2021) and Bi_4_O_5_I_2_ (Liu et al., 2014; Liu et al., 2016; Xiong et al., 2017; Xiong et al., 2018; Zhao et al., 2021) were reported.

### 4.2 Removal of radioactive iodine in synthetic groundwater

Synthetic groundwater (SGW) refers to the groundwater at the Hanford Site (WA, United States), where multiple radionuclides and other hazardous contaminants are detected. Pearce et al. (2020), Lawter et al. (2021), and Cordova et al. (2020) tested different forms of bismuth (oxy)hydroxide and bismuth subnitrate to absorb iodine (mainly IO_3_^−^) in SGW, and the results were concluded in Table 2. Except for BIN-S (silica substrate unwashed), other materials adsorbed substantially all of the iodine in the aqueous solution, which showed excellent application prospects.

**TABLE 2:** Batch experiments of some Bi-based materials in synthetic groundwater.

Material	Form	Initial concentration (mg/L)	Final concentration (mg/L)	Sampling time (d)	Iodine loading (mg/g)	K_d_ (mL/g)	Ref.
BIN^a^	powder	1.0100	0.001	1	/	2.02 × 10^5^	Pearce et al. (2020)
Bi-Co-Al	powder	0.9900	0.02	1	/	1.28 × 10^4^	
BIN	powder	0.2080	<0.126^c^	30	/	/	Lawter et al. (2021)
BIN	powder	1.0700	0.00001	1	0.214	2.14 × 10^7^	Cordova et al. (2020)
BSN^b^	powder	1.0700	0.00001		0.214	2.14 × 10^7^	
BIN-S	silica substrate (unwashed)	1.0750	0.0332		0.209	6.50 × 10^3^	
BIN	PAN beads	1.0800	0.0005		0.216	4.75 × 10^5^	
BSN	PAN beads	1.0050	0.001		0.199	1.99 × 10^5^	

^a^ BIN, is the abbreviation of Bismuth(oxy)hydroxide.

^b^ BSN, is the abbreviation of Bismuth subnitrate.

^c^ The iodine concentration is below the detection limit of the instrument.

## 4.3 Materials

A nitrate-containing bismuth compound, Bi_5_(NO_3_)O_7_, was discussed for removing and solidifying aqueous iodine among other halogenide ions from solution. The NO_3_^−^ ion was significant for the removal for its effective exchange with iodide ion. When 244 mg of Bi_5_(NO_3_)O_7_ reacted with 0.1 mol/dm^3^ of iodide at 50°C and 75°C, over 99.99% of iodine ion was removed (Kodama, 1994). However, the reaction rate was very slow at 25°C, and the influence of pH, temperature, and solution to solid ratio has not yet been studied. These preliminary results only provided a starting point for further researches.

Another nitrate incorporated bismuth oxide, BiPbO_2_NO_3_ (BPN), was developed to remove iodide ions in a solution and fix them in the solidified material BiPbO_2_I (BPI) by the ion exchange reaction (Amaya et al., 2000). An anion exchange capacity of 1.0 mEq/g and a distribution coefficient of larger than 0.1 m^3^/kg were obtained in solution at pH between 9 and 13. Takayuki (Soelberg et al., 2013) studied the leach resistance of BPI, in which the incorporation of lead helped to form strong interaction. It was concluded that BPI would be much more stable than AgI when interchanged with anions so that BPI waste can be stored for a long term without detaching the iodine. Nevertheless, same as Bi_5_(NO_3_)O_7_, the loading capacity was low as the adsorption depends on the amounts of nitrates in the compound. Basic bismuth nitrate (BBN) crystals for iodide capture were prepared by Ng and Fan (2016). The shape of BBN could be controlled through modulation of the effect of the shape-directing agent 2,3-bis(2-pyridyl)pyrazine, and showed influence on the reaction rates. The reaction rates of the Reuleaux triangledisks were faster than the other two shapes

Lee and Takahashi (2020), Pearce et al. (2020) prepared bismuth-impregnated layered mixed metal oxide (MMO) and bismuth-loaded layered double hydroxide (BiCoAl-LDH). Except for adsorption tests and characterization, the Langmuir and pseudo-second-order kinetic models were fitted to the adsorption isotherms and the adsorption kinetics, respectively. Further investigation should focus on their thermal and chemical stability for application in an industrial environment with high temperature and acidic condition.

Fiber materials are widely used in the field of adsorption thanks to their excellent surface area, porous nature, and the presence of the functional group. By varieties of modifications, these materials have improved the selectivity to increase the capture capacity of iodine. Several polyacrylonitrile were modified with bismuth oxyhydroxide (PAN-BIN) (Cordova et al., 2020), cellulose-based hybrid material (FL-δ-Bi_2_O_3_) (Xiong et al., 2017), and nano-cellulose hydrogel coated titanate-bismuth oxide membrane (CH-TBM) (Hrizi et al., 2011). The XRD analysis and Raman spectrum suggested the main product generated by chemical adsorption is Bi_4_O_5_I_2_, which was different from other materials.

With low solubility in water, Bi_2_O_3_ could capture iodine by reacting with I^−^ to form insoluble compounds, such as BiOI, Bi_5_O_7_I, and Bi_7_I_3_O_9_ (Klimakov et al., 1974; Krämer, 1979; Kodama, 1992). Wang et al. (2020) activated Bi_2_O_3_ by milling to create more oxygen vacancy and found the adsorption capacity was extremely affected by pH and temperature. Other researchers prepared flowerlike nanostructured bismuth oxide (Liu et al., 2016) and fabricated microrosette-like-δ-Bi_2_O_3_ (Liu et al., 2014). The removal capacity was higher than untreated Bi_2_O_3_, indicating the effectiveness of structural modification. In order to achieve a high specific surface area and reduced crystalline size, Zhang and Jaroniec (2017) developed mesoporous δ-Bi_2_O_3_ with the templates of SBA-15. The removal capacity of mesoporous δ-Bi_2_O_3_ doubled the same compound in another study (Zhang et al., 2006) due to the nurtured porosity of the material. The Bi_2_O_3_ without other components showed better stability in acidic conditions than inorganic materials and fiber materials.

Han et al. (2019) developed graphene oxide modified-bismuth (Bi-GO) for iodide and iodate removal in solution and compared it with Ag-Z. The adsorption capacity for IO_3_^−^ and I^−^ reached about 200-230 mg/g with more than 90% of iodate and iodide captured. Xu et al. (2019) modified the Iron-metal-organic framework (Fe-MOF) with bismuth, and the maximum capture capacities of Bi@MIL reached 96.7 mg/g.

Most Bi-based materials for capturing aqueous iodine in batch experiments had been listed by Tesfay Reda and Muhire (Tesfay Reda et al., 2021a; Muhire et al., 2022), so only the materials from the past 2 years were concluded in Table 3. Zhao developed a kind of polymer composite beads embedded by flower-like δ-Bi_2_O_3_ (δ-Bi_2_O_3_@PES), and the adsorption capacities for I^−^ and IO_3_^−^ could reach 95.4 mg/g and 170.6 mg/g, respectively. In other experiments, I_2_ is dissolved in organic solvents, and the adsorption capacity of different materials is between 180.23 mg/g to 532.9 mg/g. In the groundwater, iodine elements are comprised of 45%–84% iodate (with 15%–40% organo-iodine and <4% iodide) (Zhang et al., 2013), which requires further exploration of the above results in practical significance.

**TABLE 3:** Recent Bi-based materials for iodine capture in solution.

Material	Form of iodine	Solvent	Temperature (°C)	Capture capacity (mg/g)	Ref.
Bi_15_/Al-DMAPS	I_2_	toluene	50	251.3	Alsalbokh et al. (2021)
Bi_2_S_3_@ZIF-8	I_2_	cyclohexane	R. T.	532.9	Chen et al. (2022)
NaY-NH_4_F-Bi_2_S_3_ (30%)	I_2_	cyclohexane	/	285	Jiang et al. (2022)
δ-Bi_2_O_3_@PES	I^−^	water	R. T.	95.4	Zhao et al. (2021)
	IO_3_^−^		R. T.	170.6	
ZIF-67/CuBi–CO_3_-LDH	I_2_	n-hexane	/	180.23	Yu F et al. (2020)

## 5 Conclusion

With advantages of safety, green emission, and high economic efficiency, nuclear energy is one of the main energy sources in the world. The radioactive iodine and its compounds from serious nuclear accidents and the reprocessing of spent nuclear fuels demand efficient capture and safe storage. Compared with other mature adsorbents (such as activated carbon and Ag-based materials), Bi-based materials with lower toxicity, lower cost, and almost no radioactivity have attracted the researchers’ attention recently. These materials were applied in the capture of both aqueous iodine and vapor iodine.

The capture capacities of many Bi-based materials were higher than commercial adsorbents. Most Bi-based modified absorbents combined the advantages of high specific surface area and abundant active sites. It demonstrates that developing Bi-based modified materials based on existing supports is feasible. By characterization, leaching experiments, and immobilization experiments, it is indicated that Bi, Bi_2_O_3,_ and Bi_2_S_3_ can react with iodine to generate Bi_x_O_y_I_z_. At higher temperature, the stability of Bi_5_O_7_I is superior to BiOI and BiI_3_, which means that chemisorbed iodine can be released and additional bismuth is needed for immobilization. Although the types of Bi-based materials are rich, there is a lack of systematic research and comparison. Besides, the research about the capture of Bi-based materials for CH_3_I, one of the radioactive iodine components, needs to be further investigated. For already developed adsorbents, granulation and dynamic adsorption experiments are also of consequence.

## References

[B1] Abney C. W. Nan Y. Tavlarides L. L. (2017). X-ray absorption spectroscopy investigation of iodine capture by silver-exchanged mordenite. Ind. Eng. Chem. Res. 56 (16), 4837–4846. 10.1021/acs.iecr.7b00233

[B2] Al-Mamoori A. Alsalbokh M. Lawson S. Rownaghi A. A. Rezaei F. (2020). Development of bismuth-mordenite adsorbents for iodine capture from off-gas streams. Chem. Eng. J. 391, 123583. 10.1016/j.cej.2019.123583

[B3] Alsalbokh M. Fakeri N. Rownaghi A. A. Ludlow D. Rezaei F. (2021). Aminosilane-grafted bismuth-alumina adsorbents: Role of amine loading and bismuth content in iodine immobilization from aqueous solutions. Chem. Eng. J. 409, 128277. 10.1016/j.cej.2020.128277

[B4] Amaya T. Mukunoki A. Shibuya M. Kodama H. (2000). Study of BiPbO_2_NO_3_ for I-129 fixation under reducing conditions. MRS Online Proc. Libr. 663, 43. 10.1557/PROC-663-43

[B5] Ampelogova N. I. Kritskii V. G. Krupennikova N. I. Skvortsov A. I. (2002). Carbon-fiber adsorbent materials for removing radioactive iodine from gases. At. Energy 92 (4), 336–340. 10.1023/a:1016558127710

[B6] Aspromonte S. G. Mizrahi M. D. Schneeberger F. A. López J. M. R. Boix A. V. (2013). Study of the nature and location of silver in Ag-exchanged mordenite catalysts. Characterization by spectroscopic techniques. J. Phys. Chem. C 117 (48), 25433–25442. 10.1021/jp4046269

[B7] Bennett T. D. Saines P. J. Keen D. A. Tan J.-C. Cheetham A. K. (2013). Ball-milling-induced amorphization of zeolitic imidazolate frameworks (ZIFs) for the irreversible trapping of iodine. Chem.-Eur. J. 19 (22), 7049–7055. 10.1002/chem.201300216 23576441

[B8] Bruffey S. H. Jubin R. T. Jordan J. A. (2016). Capture of elemental and organic iodine from dilute gas streams by silver-exchanged mordenite. Procedia Chem. 21, 293–299. 10.1016/j.proche.2016.10.041

[B9] Chabauty A. L. Campayo L. Méar F. O. Montagne L. (2019). Niobium- and bismuth-silver phosphate glasses for the conditioning of radioactive iodine. J. Non-Cryst. Solids 510, 51–61. 10.1016/j.jnoncrysol.2019.01.015

[B10] Chang S. Wang K. Gao L. Liu J. Wang L. Li Y. (2022a). Highly efficient adsorption of radioiodine by a three-dimensional ordered macroporous bismuth-silica composite aerogel. Chem. Eng. Sci. 260, 117856. 10.1016/j.ces.2022.117856

[B11] Chang S. Wang K. Wang L. Song X. Liu J. Chen J. (2022b). Effects of calcination rate and temperature on microstructure and gaseous iodine capture capacity of 3DOM-SiO_2_ aerogels. Prog. Nucl. Energy 151, 104328. 10.1016/j.pnucene.2022.104328

[B12] Chapman K. W. Chupas P. J. Nenoff T. M. (2010). Radioactive iodine capture in silver-containing mordenites through nanoscale silver iodide formation. J. Am. Chem. Soc. 132 (26), 8897–8899. 10.1021/ja103110y 20550110

[B13] Chebbi M. Azambre B. Cantrel L. Huvé M. Albiol T. (2017a). Influence of structural, textural and chemical parameters of silver zeolites on the retention of methyl iodide. Microporous Mesoporous Mat. 244, 137–150. 10.1016/j.micromeso.2017.02.056

[B14] Chebbi M. Azambre B. Volkringer C. Loiseau T. (2018). Dynamic sorption properties of metal-organic frameworks for the capture of methyl iodide. Microporous Mesoporous Mat. 259, 244–254. 10.1016/j.micromeso.2017.10.018

[B15] Chebbi M. Chibani S. Paul J.-F. Cantrel L. Badawi M. (2017b). Evaluation of volatile iodine trapping in presence of contaminants: A periodic dft study on cation exchanged-faujasite. Microporous Mesoporous Mat. 239, 111–122. 10.1016/j.micromeso.2016.09.047

[B16] Chee T.-S. Tian Z. Zhang X. Lei L. Xiao C. (2020). Efficient capture of radioactive iodine by a new bismuth-decorated electrospinning carbon nanofiber. J. Nucl. Mater. 542, 152526. 10.1016/j.jnucmat.2020.152526

[B17] Chen K. Wang P. Gu A. Djam Miensah E. Gong C. Mao P. (2022). Core-shell Bi_2_S_3_ nanorods loaded ZIF-8 nanocomposites for efficient and reversible capture of radioactive iodine. Microporous Mesoporous Mat. 339, 111983. 10.1016/j.micromeso.2022.111983

[B18] Cheng Q. Yang W. Li Z. Zhu Q. Chu T. He D. (2015). Adsorption of gaseous radioactive iodine by Ag/13X zeolite at high temperatures. J. Radioanal. Nucl. Chem. 303 (3), 1883–1889. 10.1007/s10967-014-3736-3

[B19] Chibani S. Chebbi M. Lebègue S. Cantrel L. Badawi M. (2016). Impact of the Si/Al ratio on the selective capture of iodine compounds in silver-mordenite: A periodic DFT study. Phys. Chem. Chem. Phys. 18 (36), 25574–25581. 10.1039/c6cp05015h 27722672

[B20] Chien C.-C. Huang Y.-P. Wang W.-C. Chao J.-H. Wei Y.-Y. (2011). Efficiency of moso bamboo charcoal and activated carbon for adsorbing radioactive iodine. Clean-Soil Air Water 39 (2), 103–108. 10.1002/clen.201000012

[B21] Choi B.-S. Park G.-I. Lee J.-W. Yang H.-Y. Ryu S.-K. (2003). Preparation and structural studies of organotin(IV) complexes formed with organic carboxylic acids. J. Radioanal. Nucl. Chem. 256 (1), 19–26. 10.1023/a:1023383505788

[B22] Choi B. S. Park G. I. Kim J. H. Lee J. W. Ryu S. K. (2001). Adsorption equilibrium and dynamics of methyl iodide in a silver ion-exchanged zeolite column at high temperatures. Adsorpt.-J. Int. Adsorpt. Soc. 7 (2), 91–103. 10.1023/a:1011660121182

[B23] Cordova E. A. Garayburu-Caruso V. Pearce C. I. Cantrell K. J. Morad J. W. Gillispie E. C. (2020). Hybrid sorbents for ^129^I capture from contaminated groundwater. ACS Appl. Mater. Interfaces 12 (23), 26113–26126. 10.1021/acsami.0c01527 32421326

[B24] Deitz V. R. (1987). Interaction of radioactive iodine gaseous species with nuclear-grade activated carbons. Carbon 25 (1), 31–38. 10.1016/0008-6223(87)90037-6

[B25] Deuber H. (1986). Investigations on the retention of elemental radioiodine by activated carbons at high-temperatures. Nucl. Technol. 72 (1), 44–48. 10.13182/nt86-a33751

[B26] Ding Y.-H. Zhang X.-L. Zhang N. Zhang J.-Y. Zhang R. Liu Y.-F. (2018). A visible-light driven Bi_2_S_3_@ZIF-8 core–shell heterostructure and synergistic photocatalysis mechanism. Dalton Trans. 47 (3), 684–692. 10.1039/c7dt03256k 29099525

[B27] Ding Y. Fan W. Xian Q. Dan H. Zhu L. Duan T. (2023). Capture of iodine gas by Bi^0^ modified silica with different morphologies: Influence of pore characteristic on the stable and unstable forms of adsorption. Chem. Eng. J. 451, 138887. 10.1016/j.cej.2022.138887

[B28] Dinh T. D. Zhang D. Tuan V. N. (2020). High iodine adsorption performances under off-gas conditions by bismuth-modified ZnAl-LDH layered double hydroxide. RSC Adv. 10 (24), 14360–14367. 10.1039/d0ra00501k 35498468 PMC9051909

[B29] Funabashi K. Fukasawa T. Kikuchi M. (1995). Investigation of silver-impregnated alumina for removal of radioactive methyl iodide. Nucl. Technol. 109 (3), 366–372. 10.13182/nt95-a35085

[B30] Garino T. J. Nenoff T. M. Krumhansl J. L. Rademacher D. X. (2011). Low-temperature sintering Bi–Si–Zn-Oxide glasses for use in either glass composite materials or core/shell ^129^I waste forms. J. Am. Ceram. Soc. 94 (8), 2412–2419. 10.1111/j.1551-2916.2011.04542.x

[B31] Goldsmith J. R. Grossman C. M. Morton W. E. Nussbaum R. H. Kordysh E. A. Ouastel M. R. (1999). Juvenile hypothyroidism among two populations exposed to radioiodine. Environ. Health Perspect. 107 (4), 303–308. 10.1289/ehp.99107303 10090710 PMC1566525

[B32] Gonzalez-Garcia C. M. Roman S. Gonzalez J. F. Sabio E. Ledesma B. (2013). Surface free energy analysis of adsorbents used for radioiodine adsorption. Appl. Surf. Sci. 282, 714–717. 10.1016/j.apsusc.2013.06.040

[B33] Grossman C. M. Morton W. E. Nussbaum R. H. (1996). Hypothyroidism and spontaneous abortions among Hanford, Washington, downwinders. Arch. Environ. Health 51 (3), 175–176. 10.1080/00039896.1996.9936012 8687236

[B34] Grossman C. M. Nussbaum R. H. Nussbaum F. D. (2003). Cancers among residents downwind of the Hanford, Washington, plutonium production site. Arch. Environ. Health 58 (5), 267–274. 10.3200/aeoh.58.5.267-274 14738272

[B35] Grossman C. M. Nussbaum R. H. Nussbaum F. D. (2002). Thyrotoxicosis among Hanford, Washington, downwinders: A community-based health survey. Arch. Environ. Health 57 (1), 9–15. 10.1080/00039890209602911 12071367

[B36] Han S. Um W. Kim W.-S. (2019). Development of bismuth-functionalized graphene oxide to remove radioactive iodine. Dalton Trans. 48 (2), 478–485. 10.1039/c8dt03745k 30520479

[B37] He X. Cheng W. Yan M. Song W. Liu Y. Zhang Z. (2022). Performance research and engineering application of B_2_O_3_-Bi_2_O_3_-ZnO glass powder for solidifying iodine-containing silver silica gel. J. Non-Cryst. Solids 576, 121305. 10.1016/j.jnoncrysol.2021.121305

[B38] Herdes C. Prosenjak C. Román S. Müller E. A. (2013). Fundamental studies of methyl iodide adsorption in DABCO impregnated activated carbons. Langmuir 29 (23), 6849–6855. 10.1021/la401334d 23679202

[B39] Horner D. E. Mailen J. C. Posey F. A. (1977). Electrolytic trapping of iodine from process gas streams. Washington, DC: U.S. Patent and Trademark Office. U.S. Patent No 4,004,993.

[B40] Hrizi C. Samet A. Abid Y. Chaabouni S. Fliyou M. Koumina A. (2011). Crystal structure, vibrational and optical properties of a new self-organized material containing iodide anions of bismuth(III), [C_6_H_4_(NH_3_)_2_]_2_Bi_2_I_10_·4H_2_O. J. Mol. Struct. 992 (1), 96–101. 10.1016/j.molstruc.2011.02.051

[B41] Huang R. J. Seitz K. Buxmann J. Pöhler D. Hornsby K. E. Carpenter L. J. (2010). *In situ* measurements of molecular iodine in the marine boundary layer: The link to macroalgae and the implications for O-3, IO, OIO and NO_x_ . Atmos. Chem. Phys. 10 (10), 4823–4833. 10.5194/acp-10-4823-2010

[B42] Hughes J. T. Sava D. F. Nenoff T. M. Navrotsky A. (2013). Thermochemical evidence for strong iodine chemisorption by ZIF-8. J. Am. Chem. Soc. 135 (44), 16256–16259. 10.1021/ja406081r 24147801

[B43] Inagaki Y. Imamura T. Idemitsu K. Arima T. Kato O. Nishimura T. (2008). Aqueous dissolution of silver iodide and associated iodine release under reducing conditions with FeCl_2_ solution. J. Nucl. Sci. Technol. 45 (9), 859–866. 10.1080/18811248.2008.9711487

[B44] Jiang M. Zhu L. Zhao Q. Chen G. Wang Z. Zhang J. (2022). Novel synthesis of NaY-NH_4_F-Bi_2_S_3_ composite for enhancing iodine capture. Chem. Eng. J. 443, 136477. 10.1016/j.cej.2022.136477

[B45] Jung Y.-E. Kang S.-W. Yim M.-S. (2021). Feasibility study of using Bi-mna metal–organic frameworks as adsorbents for radioiodine capture at high temperature. Ind. Eng. Chem. Res. 60 (16), 5964–5975. 10.1021/acs.iecr.1c00450

[B46] Kang S. W. Yang J.-H. Yim M.-S. (2020). Examining practical application feasibility of bismuth-embedded SBA-15 for gaseous iodine adsorption. Nucl. Technol. 206 (10), 1593–1606. 10.1080/00295450.2020.1713680

[B47] Kikuchi M. Kitamura M. Yusa H. Horiuchi S. (1978). Removal of radioactive methyl iodide by silver impregnated alumina and zeolite. Nucl. Eng. Des. 47 (2), 283–287. 10.1016/0029-5493(78)90071-7

[B48] Kindel O. Hoeflich V. Herrmann F. J. Patzelt P. (1993). Removal of iodooraganic compounds from kerosene in nuclear fuel reprocessing. J. Radioanal. Nucl. Chem. 176 (3), 251–259. 10.1007/bf02163676

[B49] Klimakov A. M. Popovkin B. A. Novoselova A. V. (1974). T-X projection of structural diagrams of BiL_3_-Bi_2_O_3_ system. Russ. J. Inorg. Chem. 19, 2553–2556.

[B50] Kodama H. (1992). Solidification of iodide ion by reaction with Bi_2_O_3_ . Bull. Chem. Soc. Jpn. 65 (11), 3011–3014. 10.1246/bcsj.65.3011

[B51] Kodama H. (1994). The removal and solidification of halogenide ions using a new inorganic compound. Bull. Chem. Soc. Jpn. 67 (7), 1788–1791. 10.1246/bcsj.67.1788

[B52] Kosaka K. Asami M. Kobashigawa N. Ohkubo K. Terada H. Kishida N. (2012). Removal of radioactive iodine and cesium in water purification processes after an explosion at a nuclear power plant due to the Great East Japan Earthquake. Water Res. 46 (14), 4397–4404. 10.1016/j.watres.2012.05.055 22717151

[B53] Krämer V. (1979). Investigations of sulphide systems by thermal analysis and chemical vapour transport. J. Therm. Anal. 16 (2), 295–306. 10.1007/bf01910692

[B54] Krumhansl J. L. Nenoff T. M. (2011). Hydrotalcite-like layered bismuth–iodine–oxides as waste forms. Appl. Geochem. 26 (1), 57–64. 10.1016/j.apgeochem.2010.11.003

[B55] Kulyukhin S. A. Mizina L. V. Zanina E. V. Rumer I. A. Konovalova N. A. Levushkin D. S. (2012). Synthesis of sorbents based on coarsely dispersed silica gel, containing nanoparticles of Ag compounds, for localization of volatile radioactive iodine compounds from the water vapor-air medium. Radiochemistry 54 (4), 368–378. 10.1134/s1066362212040108

[B56] Lawter A. R. Levitskaia T. G. Qafoku O. Bowden M. E. Colon F. C. Qafoku N. P. (2021). Simultaneous immobilization of aqueous co-contaminants using a bismuth layered material. J. Environ. Radioact. 237, 106711. 10.1016/j.jenvrad.2021.106711 34388522

[B57] Lee S.-H. Takahashi Y. (2020). Selective immobilization of iodide onto a novel bismuth-impregnated layered mixed metal oxide: Batch and EXAFS studies. J. Hazard. Mater. 384, 121223. 10.1016/j.jhazmat.2019.121223 31628058

[B58] Lee U. Kim M. J. Kim H. R. (2018). Radioactive iodine analysis in environmental samples around nuclear facilities and sewage treatment plants. Nucl. Eng. Technol. 50 (8), 1355–1363. 10.1016/j.net.2018.07.017

[B59] Lee W. E. Ojovan M. I. Stennett M. C. Hyatt N. C. (2006). Immobilisation of radioactive waste in glasses, glass composite materials and ceramics. Adv. Appl. Ceram. 105 (1), 3–12. 10.1179/174367606x81669

[B60] Lin C.-C. (1981). Volatility of iodine in dilute aqueous solutions. J. Inorg. Nucl. Chem. 43 (12), 3229–3238. 10.1016/0022-1902(81)80094-2

[B61] Lin G. Zhu L. Duan T. Zhang L. Liu B. Lei J. (2019). Efficient capture of iodine by a polysulfide-inserted inorganic NiTi-layered double hydroxides. Chem. Eng. J. 378, 122181. 10.1016/j.cej.2019.122181

[B62] Liu L. Liu W. Zhao X. Chen D. Cai R. Yang W. (2014). Selective capture of iodide from solutions by microrosette-like δ-Bi_2_O_3_ . ACS Appl. Mater. Interfaces 6 (18), 16082–16090. 10.1021/am504000n 25170974

[B63] Liu S. Kang S. Wang H. Wang G. Zhao H. Cai W. (2016). Nanosheets-built flowerlike micro/nanostructured Bi_2_O_2.33_ and its highly efficient iodine removal performances. Chem. Eng. J. 289, 219–230. 10.1016/j.cej.2015.12.101

[B64] Liu S. Zeng Y. Liu J. Li J. Peng H. Xie H. (2022). Efficient capture and stable storage of radioactive iodine by bismuth-based ZIF-8 derived carbon materials as adsorbents. Sep. Purif. Technol. 302, 122151. 10.1016/j.seppur.2022.122151

[B65] Liu Y. Li B. Shu X. Zhang Z. Wei G. Liu Y. (2021). Low-sintering-temperature borosilicate glass to immobilize silver-coated silica-gel with different iodine loadings. J. Hazard. Mater. 403, 123588. 10.1016/j.jhazmat.2020.123588 32777747

[B66] Ma S. Islam S. M. Shim Y. Gu Q. Wang P. Li H. (2014). Highly efficient iodine capture by layered double hydroxides intercalated with polysulfides. Chem. Mater. 26 (24), 7114–7123. 10.1021/cm5036997

[B67] Mailen J. C. Horner D. E. (1976). Removal of radioiodine from gas streams by electrolytic scrubbing. Nucl. Technol. 30 (3), 317–324. 10.13182/nt76-a31646

[B68] Michel R. Handl J. Ernst T. Botsch W. Szidat S. Schmidt A. (2005). Iodine-129 in soils from Northern Ukraine and the retrospective dosimetry of the iodine-131 exposure after the Chernobyl accident. Sci. Total Environ. 340 (1), 35–55. 10.1016/j.scitotenv.2004.08.006 15752491

[B69] Miensah E. D. Gu A. Kokuloku L. T. Jr Chen K. Wang P. Gong C. (2022). Strategies for radioiodine capture by metal organic frameworks and their derived materials. Microporous Mesoporous Mat. 341, 112041. 10.1016/j.micromeso.2022.112041

[B70] Mineo H. Gotoh M. Iizuka M. Fujisaki S. Hagiya H. Uchiyama G. (2003). Applicability of a model predicting iodine-129 profile in a silver nitrate silica-gel column for dissolver off-gas treatment of fuel reprocessing. Sep. Sci. Technol. 38 (9), 1981–2001. 10.1081/ss-120020130

[B71] Mineo H. Gotoh M. Iizuka M. Fujisaki S. Uchiyama G. (2002). A simple model predicting iodine profile in a packed bed of silica-gel impregnated with silver nitrate. J. Nucl. Sci. Technol. 39 (3), 241–247. 10.1080/18811248.2002.9715181

[B72] Modolo G. Odoj R. (1997). Investigations on the partitioning of ^129^I from silver-impregnated silica in preparation for future transmutation. Nucl. Technol. 117 (1), 80–86. 10.13182/nt97-a35337

[B73] Moore R. C. Pearce C. I. Morad J. W. Chatterjee S. Levitskaia T. G. Asmussen R. M. (2020). Iodine immobilization by materials through sorption and redox-driven processes: A literature review. Sci. Total Environ. 716, 132820. 10.1016/j.scitotenv.2019.06.166 31982189

[B74] Moriyama K. Tashiro S. Chiba N. Hirayama F. Maruyama Y. Nakamura H. (2010). Experiments on the release of gaseous iodine from gamma-irradiated aqueous CsI solution and influence of oxygen and methyl isobutyl ketone (MIBK). J. Nucl. Sci. Technol. 47 (3), 229–237. 10.1080/18811248.2010.9711949

[B75] Mowry C. D. Brady P. V. Garino T. J. Nenoff T. M. (2015). Development and durability testing of a low-temperature sintering Bi–Si–Zn oxide glass composite material (GCM) ^129^I Waste Form. J. Am. Ceram. Soc. 98 (10), 3094–3104. 10.1111/jace.13751

[B76] Muhire C. Tesfay Reda A. Zhang D. Xu X. Cui C. (2022). An overview on metal oxide-based materials for iodine capture and storage. Chem. Eng. J. 431, 133816. 10.1016/j.cej.2021.133816

[B77] Munakata K. Kanjo S. Yamatsuki S. Koga A. Ianovski D. (2003). Adsorption of noble gases on silver-mordenite. J. Nucl. Sci. Technol. 40 (9), 695–697. 10.1080/18811248.2003.9715408

[B78] Nan Y. Liu J. Tang S. Lin R. Tavlarides L. L. (2018). Silver-exchanged mordenite for capture of water vapor in off-gas streams: A study of adsorption kinetics. Ind. Eng. Chem. Res. 57 (3), 1048–1058. 10.1021/acs.iecr.7b04420

[B79] Nandanwar S. U. Coldsnow K. Utgikar V. Sabharwall P. Eric Aston D. (2016). Capture of harmful radioactive contaminants from off-gas stream using porous solid sorbents for clean environment – a review. Chem. Eng. J. 306, 369–381. 10.1016/j.cej.2016.07.073

[B80] Nenoff T. M. Rodriguez M. A. Soelberg N. R. Chapman K. W. (2014). Silver-mordenite for radiologic gas capture from complex streams: Dual catalytic CH_3_I decomposition and I confinement. Microporous Mesoporous Mat. 200, 297–303. 10.1016/j.micromeso.2014.04.041

[B81] Ng C. H. B. Fan W. Y. (2016). Shape-controlled preparation of basic bismuth nitrate crystals with high iodide-removal capacities. ChemNanoMat 2 (2), 133–139. 10.1002/cnma.201500179

[B82] Ojovan M. I. Lee W. E. (2011). Glassy wasteforms for nuclear waste immobilization. Metall. Mater. Trans. A-Phys. Metall. Mater. Sci. 42 (4), 837–851. 10.1007/s11661-010-0525-7

[B83] Pearce C. I. Cordova E. A. Garcia W. L. Saslow S. A. Cantrell K. J. Morad J. W. (2020). Evaluation of materials for iodine and technetium immobilization through sorption and redox-driven processes. Sci. Total Environ. 716, 136167. 10.1016/j.scitotenv.2019.136167 31955840

[B84] Pei C. Ben T. Xu S. Qiu S. (2014). Ultrahigh iodine adsorption in porous organic frameworks. J. Mater. Chem. A 2 (20), 7179–7187. 10.1039/c4ta00049h

[B85] Pham T. C. T. Docao S. Hwang I. C. Song M. K. Choi D. Y. Moon D. (2016). Capture of iodine and organic iodides using silica zeolites and the semiconductor behaviour of iodine in a silica zeolite. Energy Environ. Sci. 9 (3), 1050–1062. 10.1039/c5ee02843d

[B86] Pillar E. A. Guzman M. I. Rodriguez J. M. (2013). Conversion of iodide to hypoiodous acid and iodine in aqueous microdroplets exposed to ozone. Environ. Sci. Technol. 47 (19), 10971–10979. 10.1021/es401700h 23987087

[B87] Pires J. Carvalho A. de Carvalho M. B. (2001). Adsorption of volatile organic compounds in Y zeolites and pillared clays. Microporous Mesoporous Mat. 43 (3), 277–287. 10.1016/s1387-1811(01)00207-4

[B88] Qin H. Lv Y. Kobayashi H. Xiao M. Song H. Yang J. (2022). Fabrication of NOTT-220 @I_2_ via iodine adsorption and immobilization in bismuth organic framework for efficient CO_2_ photo-reduction. J. Alloys Compd. 920, 165900. 10.1016/j.jallcom.2022.165900

[B89] Ranjan M. Singh P. K. Srivastav A. L. (2020). A review of bismuth-based sorptive materials for the removal of major contaminants from drinking water. Environ. Sci. Pollut. Res. 27 (15), 17492–17504. 10.1007/s11356-019-05359-9 31172431

[B90] Riley B. J. Chun J. Ryan J. V. Matyas J. Li X. S. Matson D. W. (2011). Chalcogen-based aerogels as a multifunctional platform for remediation of radioactive iodine. RSC Adv. 1 (9), 1704–1715. 10.1039/c1ra00351h

[B91] Riley B. J. Chun J. Um W. Lepry W. C. Matyas J. Olszta M. J. (2013). Chalcogen-based aerogels as sorbents for radionuclide remediation. Environ. Sci. Technol. 47 (13), 7540–7547. 10.1021/es400595z 23763706

[B92] Riley B. J. Vienna J. D. Strachan D. M. McCloy J. S. Jerden J. L. (2016). Materials and processes for the effective capture and immobilization of radioiodine: A review. J. Nucl. Mater. 470, 307–326. 10.1016/j.jnucmat.2015.11.038

[B93] Sakurai T. Takahashi A. (1994). Catalytic effect of silver-impregnated silica-gel (AgS) on reaction of methyl iodide with nitrogen dioxide. J. Nucl. Sci. Technol. 31 (1), 86–87. 10.1080/18811248.1994.9735119

[B94] Sava D. F. Chapman K. W. Rodriguez M. A. Greathouse J. A. Crozier P. S. Zhao H. (2013). Competitive I_2_ sorption by Cu-BTC from humid gas streams. Chem. Mater. 25 (13), 2591–2596. 10.1021/cm401762g

[B95] Sava D. F. Garino T. J. Nenoff T. M. (2012). Iodine confinement into Metal–Organic Frameworks (MOFs): Low-temperature sintering glasses to form novel glass composite material (GCM) alternative waste forms. Ind. Eng. Chem. Res. 51 (2), 614–620. 10.1021/ie200248g

[B96] Sava D. F. Rodriguez M. A. Chapman K. W. Chupas P. J. Greathouse J. A. Crozier P. S. (2011). Capture of volatile iodine, a gaseous fission product, by zeolitic imidazolate framework-8. J. Am. Chem. Soc. 133 (32), 12398–12401. 10.1021/ja204757x 21766858

[B97] Sava Gallis D. F. Ermanoski I. Greathouse J. A. Chapman K. W. Nenoff T. M. (2017). Iodine gas adsorption in nanoporous materials: A combined experiment modeling study. Ind. Eng. Chem. Res. 56 (8), 2331–2338. 10.1021/acs.iecr.6b04189

[B98] Scott S. M. Hu T. Yao T. Xin G. Lian J. (2015). Graphene-based sorbents for iodine-129 capture and sequestration. Carbon 90, 1–8. 10.1016/j.carbon.2015.03.070

[B99] Soelberg N. R. Garn T. G. Greenhalgh M. R. Law J. D. Jubin R. Strachan D. M. (2013). Radioactive iodine and krypton control for nuclear fuel reprocessing facilities. Sci. Technol. Nucl. Install. 2013, 1–12. 10.1155/2013/702496

[B100] Subrahmanyam K. S. Sarma D. Malliakas C. D. Polychronopoulou K. Riley B. J. Pierce D. A. (2015). Chalcogenide aerogels as sorbents for radioactive iodine. Chem. Mater. 27 (7), 2619–2626. 10.1021/acs.chemmater.5b00413

[B101] Taghipour F. Evans G. J. (2000). Radiolytic organic iodide formation under nuclear reactor accident conditions. Environ. Sci. Technol. 34 (14), 3012–3017. 10.1021/es990507d

[B102] Takeshita K. Azegami Y. (2004). Development of thermal swing adsorption (TSA) process for complete recovery of iodine in dissolver off-gas. J. Nucl. Sci. Technol. 41 (1), 91–94. 10.1080/18811248.2004.9715463

[B103] Tanabe H. Sakuragi T. Yamaguchi K. Sato T. Owada H. (2010). Development of new waste forms to immobilize iodine-129 released from a spent fuel reprocessing plant. Adv. Sci. Technol. 73, 158–170. 10.4028/www.scientific.net/ast.73.158

[B104] Taylor D. M. (1981). The radiotoxicology of iodine. J. Radioanal. Chem. 65 (1), 195–208. 10.1007/bf02516104

[B105] Tesfay Reda A. Pan M. Zhang D. Xu X. (2021a). Bismuth-based materials for iodine capture and storage: A review. J. Environ. Chem. Eng. 9 (4), 105279. 10.1016/j.jece.2021.105279

[B106] Tesfay Reda A. Zhang D. Xu X. Pan M. Chang C. Muhire C. (2021b). Bismuth-impregnated aluminum/copper oxide-pillared montmorillonite for efficient vapor iodine sorption. Sep. Purif. Technol. 270, 118848. 10.1016/j.seppur.2021.118848

[B107] Tesfay Reda A. Zhang D. Xu X. Xu S. (2022). Highly stable iodine capture by pillared montmorillonite functionalized Bi_2_O_3_@g-C_3_N_4_ nanosheets. Sep. Purif. Technol. 292, 120994. 10.1016/j.seppur.2022.120994

[B108] Thomas G. D. Smith S. M. Turcotte J. A. (2009). Using public relations strategies to prompt populations at risk to seek health information: The Hanford Community Health Project. Health promot. Pract. 10 (1), 92–101. 10.1177/1524839907307676 18353906

[B109] Tian Z. Chee T.-S. Meng R. Hao Y. Zhou X. Ma B. (2022). Incipient wetness impregnation to prepare bismuth-modified all-silica beta zeolite for efficient radioactive iodine capture. Environ. Funct. Mater. 1 (1), 92–104. 10.1016/j.efmat.2022.05.006

[B110] Tian Z. Chee T.-S. Zhang X. Lei L. Xiao C. (2021a). Novel bismuth-based electrospinning materials for highly efficient capture of radioiodine. Chem. Eng. J. 412, 128687. 10.1016/j.cej.2021.128687

[B111] Tian Z. Chee T.-S. Zhu L. Duan T. Zhang X. Lei L. (2021b). Comprehensive comparison of bismuth and silver functionalized nickel foam composites in capturing radioactive gaseous iodine. J. Hazard. Mater. 417, 125978. 10.1016/j.jhazmat.2021.125978 34015715

[B112] Verger P. Aurengo A. Geoffroy B. Le Guen B. (2001). Iodine kinetics and effectiveness of stable iodine prophylaxis after intake of radioactive iodine: A review. Thyroid 11 (4), 353–360. 10.1089/10507250152039082 11349833

[B113] Wang C. Hu H. Yan S. Zhang Q. (2020). Activating Bi_2_O_3_ by ball milling to induce efficiently oxygen vacancy for incorporating iodide anions to form BiOI. Chem. Phys. 533, 110739. 10.1016/j.chemphys.2020.110739

[B114] Wang J. Zhuang S. (2019). Covalent organic frameworks (COFs) for environmental applications. Coord. Chem. Rev. 400, 213046. 10.1016/j.ccr.2019.213046

[B115] Wang P. Xu Q. Li Z. Jiang W. Jiang Q. Jiang D. (2018). Exceptional iodine capture in 2D covalent organic frameworks. Adv. Mater. 30 (29), 1801991. 10.1002/adma.201801991 29806216

[B116] Wei G. Luo F. Li B. Liu Y. Yang J. Zhang Z. (2021). Immobilization of iodine waste forms: A low-sintering temperature with Bi_2_O_3_-B_2_O_3_-ZnO glass. Ann. Nucl. Energy 150, 107817. 10.1016/j.anucene.2020.107817

[B117] Wei G. Shu X. Zhang Z. Li Q. Liu Y. Wang X. (2020). B_2_O_3_–Bi_2_O_3_–ZnO based materials for low-sintering temperature immobilization of iodine adsorbed waste. J. Solid State Chem. 289, 121518. 10.1016/j.jssc.2020.121518

[B118] Woo T. H. (2013). Atmospheric modeling of radioactive material dispersion and health risk in Fukushima Daiichi nuclear power plants accident. Ann. Nucl. Energy 53, 197–201. 10.1016/j.anucene.2012.09.003

[B119] Wren J. C. Ball J. M. Glowa G. A. (1999). The interaction of iodine with organic material in containment. Nucl. Technol. 125 (3), 337–362. 10.13182/NT99-A2952

[B120] Wu B. Yan M. Luo F. Shu X. Liu Y. Wei G. (2021). Low-temperature fabrication of glass-based iodine waste forms via a novel preparation method. J. Solid State Chem. 300, 122186. 10.1016/j.jssc.2021.122186

[B121] Wu D. Wei G. Shu X. Liu Y. Han W. Zhang Z. (2022). Immobilization of iodine waste at low sintering temperature: Phase evolution and microstructure transformation. Ann. Nucl. Energy 173, 109145. 10.1016/j.anucene.2022.109145

[B122] Xian Q. Chen L. Fan W. Liu Y. He X. Dan H. (2022a). Facile synthesis of novel Bi^0^-SBA-15 adsorbents by an improved impregnation reduction method for highly efficient capture of iodine gas. J. Hazard. Mater. 424, 127678. 10.1016/j.jhazmat.2021.127678 34775310

[B123] Xian Q. Gan Y. Yu J. Xiao X. Chen Q. Dan H. (2022b). Scalable and economical Bi^0^-SiO_2_ for the high efficient capture of iodine gas. J. Nucl. Mater. 567, 153849. 10.1016/j.jnucmat.2022.153849

[B124] Xiong Y. Dang B. Wang C. Wan H. Zhang S. Sun Q. (2017). Cellulose fibers constructed convenient recyclable 3D graphene-formicary-like delta-Bi_2_O_3_ aerogels for the selective capture of iodide. ACS Appl. Mater. Interfaces 9 (24), 20554–20560. 10.1021/acsami.7b03516 28570051

[B125] Xiong Y. Wang C. Wang H. Jin C. Sun Q. Xu X. (2018). Nano-cellulose hydrogel coated flexible titanate-bismuth oxide membrane for trinity synergistic treatment of super-intricate anion/cation/oily-water. Chem. Eng. J. 337, 143–151. 10.1016/j.cej.2017.12.080

[B126] Xu W. Zhang W. Kang J. Li B. (2019). Facile synthesis of mesoporous Fe-based MOFs loading bismuth with high speed adsorption of iodide from solution. J. Solid State Chem. 269, 558–565. 10.1016/j.jssc.2018.10.028

[B127] Yan M. Wu B. Luo F. Shu X. Liu Y. Wei G. (2021). Bi_2_O_3_ doped B_2_O_3_-ZnO glass powder for immobilization of radioactive iodine waste at low temperature. Ann. Nucl. Energy 161, 108480. 10.1016/j.anucene.2021.108480

[B128] Yang J. H. Cho Y.-J. Shin J. M. Yim M.-S. (2015). Bismuth-embedded SBA-15 mesoporous silica for radioactive iodine capture and stable storage. J. Nucl. Mater. 465, 556–564. 10.1016/j.jnucmat.2015.06.043

[B129] Yang J. H. Park H. S. Ahn D.-H. Yim M.-S. (2016). Glass composite waste forms for iodine confined in bismuth-embedded SBA-15. J. Nucl. Mater. 480, 150–158. 10.1016/j.jnucmat.2016.08.001

[B130] Yang K. Wang Y. Shen J. Scott S. M. Riley B. J. Vienna J. D. (2022). Cs_3_Bi_2_I_9_-hydroxyapatite composite waste forms for cesium and iodine immobilization. J. Adv. Ceram. 11 (5), 712–728. 10.1007/s40145-021-0565-z

[B131] Yang K. Zhu W. Scott S. Wang Y. Wang J. Riley B. J. (2021). Immobilization of cesium and iodine into Cs_3_Bi_2_I_9_ perovskite-silica composites and core-shell waste forms with high waste loadings and chemical durability. J. Hazard. Mater. 401, 123279. 10.1016/j.jhazmat.2020.123279 32629351

[B132] Yang Y. Xiong X. Fan Y. Lai Z. Xu Z. Luo F. (2019). Insight into volatile iodine uptake properties of covalent organic frameworks with different conjugated structures. J. Solid State Chem. 279, 120979. 10.1016/j.jssc.2019.120979

[B133] Yang Y. Zeng Z. Zhang C. Huang D. Zeng G. Xiao R. (2018). Construction of iodine vacancy-rich BiOI/Ag@AgI Z-scheme heterojunction photocatalysts for visible-light-driven tetracycline degradation: Transformation pathways and mechanism insight. Chem. Eng. J. 349, 808–821. 10.1016/j.cej.2018.05.093

[B134] Yu F. Chen Y. Wang Y. Liu C. Qin J. (2020). Synthesis of metal–organic framework nanocrystals immobilized with 3D flowerlike Cu–Bi-layered double hydroxides for iodine efficient removal. J. Mater. Res. 35 (3), 299–311. 10.1557/jmr.2020.1

[B135] Yu Q. Jiang X. Cheng Z. Liao Y. Pu Q. Duan M. (2020). Millimeter-sized Bi_2_S_3_@polyacrylonitrile hybrid beads for highly efficient iodine capture. New J. Chem. 44 (39), 16759–16768. 10.1039/d0nj03229h

[B136] Yuan Y. Dong X. Chen Y. Zhang M. (2016). Computational screening of iodine uptake in zeolitic imidazolate frameworks in a water-containing system. Phys. Chem. Chem. Phys. 18 (33), 23246–23256. 10.1039/c6cp02156e 27499079

[B137] Zakirova G. G. Mladentsev D. Y. Borisova N. E. (2017). Synthesis of chelating tertiary phosphine oxides via palladium-catalysed C–P bond formation. Tetrahedron Lett. 58 (35), 3415–3417. 10.1016/j.tetlet.2017.07.055

[B138] Zhang L. Jaroniec M. (2017). SBA-15 templating synthesis of mesoporous bismuth oxide for selective removal of iodide. J. Colloid Interface Sci. 501, 248–255. 10.1016/j.jcis.2017.04.063 28458225

[B139] Zhang L. Wang W. Yang J. Chen Z. Zhang W. Zhou L. (2006). Sonochemical synthesis of nanocrystallite Bi_2_O_3_ as a visible-light-driven photocatalyst. Appl. Catal. A 308, 105–110. 10.1016/j.apcata.2006.04.016

[B140] Zhang S. Du J. Xu C. Schwehr K. A. Ho Y. F. Li H. P. (2011). Concentration-dependent mobility, retardation, and speciation of iodine in surface sediment from the Savannah River Site. Environ. Sci. Technol. 45 (13), 5543–5549. 10.1021/es1040442 21663237

[B141] Zhang S. Xu C. Creeley D. Ho Y.-F. Li H.-P. Grandbois R. (2013). Iodine-129 and iodine-127 speciation in groundwater at the Hanford site, U.S.: Iodate incorporation into calcite. Environ. Sci. Technol. 47 (17), 9635–9642. 10.1021/es401816e 23885783

[B142] Zhang X. da Silva I. Fazzi R. Sheveleva A. M. Han X. Spencer B. F. (2019). Iodine adsorption in a redox-active metal–organic framework: Electrical conductivity induced by Host−Guest charge-transfer. Inorg. Chem. 58 (20), 14145–14150. 10.1021/acs.inorgchem.9b02176 31566954 PMC6806328

[B143] Zhao H. Nenoff T. M. Jennings G. Chupas P. J. Chapman K. W. (2011). Determining quantitative kinetics and the structural mechanism for particle growth in porous templates. J. Phys. Chem. Lett. 2 (21), 2742–2746. 10.1021/jz201260n

[B144] Zhao Q. Chen G. Wang Z. Jiang M. Lin J. Zhang L. (2021). Efficient removal and immobilization of radioactive iodide and iodate from aqueous solutions by bismuth-based composite beads. Chem. Eng. J. 426, 131629. 10.1016/j.cej.2021.131629

[B145] Zhou J. Hao S. Gao L. Zhang Y. (2014). Study on adsorption performance of coal based activated carbon to radioactive iodine and stable iodine. Ann. Nucl. Energy 72, 237–241. 10.1016/j.anucene.2014.05.028

[B146] Zou H. Guo J. Song M. Yi F. Wang X. Pan N. (2021). Bi_2_S_3_-reduced graphene oxide composite for gaseous radioiodine capture and its immobilization within glass composite material. Prog. Nucl. Energy 135, 103705. 10.1016/j.pnucene.2021.103705

[B147] Zou H. Yi F. Song M. Wang X. Bian L. Li W. (2019). Novel synthesis of Bi-Bi_2_O_3_-TiO_2_-C composite for capturing iodine-129 in off-gas. J. Hazard. Mater. 365, 81–87. 10.1016/j.jhazmat.2018.11.001 30412810

